# Supplementary material for: Exposure and risk factors for COVID-19 and the impact of staying home on Michigan residents
Source: PLoS One. 2021 Feb 8;16(2):e0246447. doi: 10.1371/journal.pone.0246447 (PMC7870003; doi:10.1371/journal.pone.0246447)
Supplement: S1 Table — (DOCX) [file pone.0246447.s001.docx]

| **Table S1**. Biorepository Studies |  | |  | |  | |  |
| --- | --- | --- | --- | --- | --- | --- | --- |
| **Cohort Name** | **HUM** | **N cases** | | **N valid emails** | | **N enrolled** | |
| Michigan Genomics Initiative-Anesthesiology Collection Effort (MGI) | HUM00071298 | 64,155 | | 42,293 | | 6,625 | |
| Cardiovascular Health Improvement Project (CHIP) | HUM00052866 | 5,254 | | 3,253 | | 537 | |
| Michigan Racial Equality and Community Health (MREACH) | HUM00146997 | 272 | | 202 | | 26 | |
| Mental Health Biobank (MHB2) | HUM00097962 | 1,975 | | 1,616 | | 259 | |
| Michigan Genetics of Atherosclerosis and Lipids (MGOAL) | HUM00095768 | 278 | | 216 | | 41 | |
| Biorepository with a Special Emphasis on COVID-19 Related Research | HUM00179787 | 85 | | 58 | | 20 | |
| Biobank to Illuminate the Genomic Basis of Pediatric Disease (BIGBiRD) | HUM00141565 | 211 | | 113 | | 13 | |
| Analgesic outcomes Study (AOS) | HUM00028683 | 781 | | 460 | | 76 | |
| Michigan Genomics Initiative-Metabolism, Endocrinology, and Diabetes (MGI-MEND) | HUM00099197 | 3,140 | | 2,496 | | 432 | |
| Bicuspid Aortic Valve Registry (BAV Registry) | HUM00035836 | 207 | | 113 | | 18 | |
