# Supplementary material for: Exposure and risk factors for COVID-19 and the impact of staying home on Michigan residents
Source: PLoS One. 2021 Feb 8;16(2):e0246447. doi: 10.1371/journal.pone.0246447 (PMC7870003; doi:10.1371/journal.pone.0246447)
Supplement: S2 Table — (DOCX) [file pone.0246447.s002.docx]

| **Table S2.** Demographic, social economic status, environmental factors, and self-reported health conditions by types of diagnosed | | | | | | | | |
| --- | --- | --- | --- | --- | --- | --- | --- | --- |
|  |  |  | **Overall** |  | **COVID-19 diagnosed type** | | |  |
|  |  |  |  |  | **Self-dx** | **Test +** | **Doc-dx** | **p** |
|  |  | n | 8167 |  | 145 | 77 | 36 |  |
| Race | |  |  |  |  |  |  |  |
|  | African American |  | 239 ( 3.1) |  | 6 ( 4.4) | 12 ( 17.1) | 1 ( 2.9) | 0.003 |
|  | European American |  | 7499 (96.9) |  | 129 ( 95.6) | 58 ( 82.9) | 33 ( 97.1) |  |
| Sex | |  |  |  |  |  |  |  |
|  | Female |  | 4743 (58.6) |  | 97 ( 66.9) | 38 ( 50.0) | 26 ( 72.2) | 0.021 |
|  | Male |  | 3354 (41.4) |  | 48 ( 33.1) | 38 ( 50.0) | 10 ( 27.8) |  |
| Age | |  |  |  |  |  |  |  |
|  |  |  | 59.09 (14.76) |  | 55.18 (12.81) | 53.21 (14.85) | 46.69 (14.76) | 0.004 |
| BMI | |  |  |  |  |  |  |  |
|  |  |  | 29.12 (6.64) |  | 29.11 (6.29) | 30.02 (7.50) | 30.05 (6.00) | 0.555 |
| BMI category | |  |  |  |  |  |  |  |
|  | underweight |  | 74 ( 0.9) |  | 1 ( 0.7) | 3 ( 4.1) | 0 ( 0.0) | 0.236 |
|  | normal |  | 2211 (28.2) |  | 40 ( 28.4) | 13 ( 17.8) | 6 ( 16.7) |  |
|  | overweight |  | 2619 (33.4) |  | 43 ( 30.5) | 25 ( 34.2) | 13 ( 36.1) |  |
|  | obese |  | 2933 (37.4) |  | 57 ( 40.4) | 32 ( 43.8) | 17 ( 47.2) |  |
| Education | |  |  |  |  |  |  |  |
|  | High school or less |  | 1390 (17.1) |  | 28 ( 19.4) | 20 ( 26.7) | 2 ( 5.6) | 0.165 |
|  | Associate degree |  | 1296 (16.0) |  | 23 ( 16.0) | 11 ( 14.7) | 5 ( 13.9) |  |
|  | Bachelor's degree |  | 2530 (31.2) |  | 45 ( 31.2) | 23 ( 30.7) | 11 ( 30.6) |  |
|  | Advanced degree |  | 2905 (35.8) |  | 48 ( 33.3) | 21 ( 28.0) | 18 ( 50.0) |  |
| Income | |  |  |  |  |  |  |  |
|  | <40 |  | 1080 (14.0) |  | 13 ( 9.3) | 9 ( 12.0) | 3 ( 8.3) | 0.747 |
|  | 40-100 |  | 3355 (43.6) |  | 64 ( 45.7) | 28 ( 37.3) | 14 ( 38.9) |  |
|  | >100 |  | 3257 (42.3) |  | 63 ( 45.0) | 38 ( 50.7) | 19 ( 52.8) |  |
| Current living situation | |  |  |  |  |  |  |  |
|  | A family member owns the house I live in |  | 471 ( 5.8) |  | 6 ( 4.1) | 5 ( 6.8) | 6 ( 16.7) | 0.025 |
|  | I own a house |  | 6651 (81.9) |  | 123 ( 84.8) | 55 ( 75.3) | 23 ( 63.9) |  |
|  | I rent a house/apartment |  | 807 ( 9.9) |  | 14 ( 9.7) | 8 ( 11.0) | 6 ( 16.7) |  |
|  | Other |  | 193 ( 2.4) |  | 2 ( 1.4) | 5 ( 6.8) | 1 ( 2.8) |  |
| Have any of your family members been diagnosed with COVID-19 within two weeks after you saw them last? | |  |  |  |  |  |  |  |
|  | Yes |  | 52 (13.4) |  | 10 ( 8.5) | 24 ( 40.7) | 5 ( 23.8) | 6.E-08 |
|  | No |  | 292 (75.3) |  | 81 ( 69.2) | 35 ( 59.3) | 16 ( 76.2) |  |
|  | Unsure |  | 44 (11.3) |  | 26 ( 22.2) | 0 ( 0.0) | 0 ( 0.0) |  |
| Has anyone outside of your household been diagnosed with COVID-19 within two weeks after you saw them last? | |  |  |  |  |  |  |  |
|  | Yes |  | 43 (11.1) |  | 7 ( 5.9) | 13 ( 26.0) | 4 ( 13.8) | 0.003 |
|  | No |  | 203 (52.2) |  | 56 ( 47.1) | 24 ( 48.0) | 15 ( 51.7) |  |
|  | Unsure |  | 143 (36.8) |  | 56 ( 47.1) | 13 ( 26.0) | 10 ( 34.5) |  |
| Approximately how many days did your symptoms last? (time from symptom onset to completely recovered) | |  |  |  |  |  |  |  |
|  |  |  | 14.69 (11.02) |  | 15.76 (10.86) | 19.17 (11.25) | 20.11 (11.75) | 0.031 |
| Please indicate your physical activity participation in relation to shelter at home recommendations. | |  |  |  |  |  |  |  |
|  | Before |  | 3.69 (1.85) |  | 3.91 (1.84) | 3.91 (1.91) | 3.71 (1.79) | 0.853 |
|  | After |  | 3.74 (2.05) |  | 3.58 (2.08) | 2.98 (2.05) | 3.10 (1.95) | 0.171 |
| What precautions are you taking to protect yourself or others from COVID-19? | |  |  |  |  |  |  |  |
|  | Frequent hand washing |  | 7849 (96.1) |  | 142 ( 97.9) | 71 ( 92.2) | 35 ( 97.2) | 0.102 |
|  | Hand disinfectant use |  | 6736 (82.5) |  | 121 ( 83.4) | 65 ( 84.4) | 31 ( 86.1) | 0.923 |
|  | Cover face while sneezing/coughing |  | 7570 (92.7) |  | 138 ( 95.2) | 70 ( 90.9) | 33 ( 91.7) | 0.429 |
|  | Wearing a mask |  | 7781 (95.3) |  | 141 ( 97.2) | 69 ( 89.6) | 36 (100.0) | 0.013 |
|  | Avoiding public transport |  | 6206 (76.0) |  | 101 ( 69.7) | 47 ( 61.0) | 28 ( 77.8) | 0.175 |
|  | Social distancing |  | 7517 (92.0) |  | 138 ( 95.2) | 66 ( 85.7) | 36 (100.0) | 0.007 |
|  | Work from home |  | 2987 (36.6) |  | 65 ( 44.8) | 21 ( 27.3) | 18 ( 50.0) | 0.018 |
|  | Avoid travel in general |  | 5670 (69.4) |  | 104 ( 71.7) | 46 ( 59.7) | 28 ( 77.8) | 0.087 |
|  | Self-Isolation |  | 2159 (26.4) |  | 40 ( 27.6) | 9 ( 11.7) | 9 ( 25.0) | 0.024 |
|  | None of these |  | 31 ( 0.4) |  | 1 ( 0.7) | 3 ( 3.9) | 0 ( 0.0) | 0.132 |
| What COVID-19 symptoms did you have? | |  |  |  |  |  |  |  |
|  | No symptoms |  | 394 (44.4) |  | 1 ( 0.7) | 6 ( 7.8) | 0 ( 0.0) | 0.005 |
|  | Fever (100.4F or more) |  | 222 (25.0) |  | 89 ( 61.4) | 45 ( 58.4) | 25 ( 69.4) | 0.531 |
|  | Fatigue |  | 382 (43.1) |  | 124 ( 85.5) | 56 ( 72.7) | 34 ( 94.4) | 0.008 |
|  | Cough |  | 328 (37.0) |  | 119 ( 82.1) | 41 ( 53.2) | 26 ( 72.2) | 3.E-05 |
|  | Runny Nose |  | 178 (20.1) |  | 66 ( 45.5) | 15 ( 19.5) | 9 ( 25.0) | 2.E-04 |
|  | Sneezing |  | 111 (12.5) |  | 34 ( 23.4) | 8 ( 10.4) | 10 ( 27.8) | 0.033 |
|  | Congestion |  | 184 (20.7) |  | 62 ( 42.8) | 22 ( 28.6) | 15 ( 41.7) | 0.107 |
|  | Loss of Smell/Taste |  | 136 (15.3) |  | 53 ( 36.6) | 30 ( 39.0) | 16 ( 44.4) | 0.678 |
|  | Shortness of Breath |  | 244 (27.5) |  | 82 ( 56.6) | 45 ( 58.4) | 28 ( 77.8) | 0.063 |
|  | Chest Pain |  | 139 (15.7) |  | 48 ( 33.1) | 19 ( 24.7) | 21 ( 58.3) | 0.002 |
|  | Muscle Aches |  | 265 (29.9) |  | 88 ( 60.7) | 47 ( 61.0) | 28 ( 77.8) | 0.147 |
|  | Nausea/Diarrhea |  | 179 (20.2) |  | 56 ( 38.6) | 33 ( 42.9) | 17 ( 47.2) | 0.599 |
|  | Headache |  | 290 (32.7) |  | 88 ( 60.7) | 45 ( 58.4) | 25 ( 69.4) | 0.524 |
|  | Sore Throat |  | 243 (27.4) |  | 80 ( 55.2) | 24 ( 31.2) | 17 ( 47.2) | 0.003 |
| Which of the following things did you do in the two weeks prior to showing symptoms? | |  |  |  |  |  |  |  |
|  | None of these things |  | 57 (11.6) |  | 9 ( 6.2) | 12 ( 16.9) | 3 ( 8.3) | 0.043 |
|  | Participate in any Festivals/Events of over 50 people |  | 70 (14.2) |  | 32 ( 22.2) | 6 ( 8.5) | 3 ( 8.3) | 0.014 |
|  | Go to the Grocery Store |  | 365 (74.0) |  | 119 ( 82.6) | 44 ( 62.0) | 29 ( 80.6) | 0.003 |
|  | Work in direct contact with the public (e.g. grocery store employee, cashier, clerk, etc.) |  | 177 (35.9) |  | 46 ( 31.9) | 33 ( 46.5) | 11 ( 30.6) | 0.087 |
|  | Travel within your State |  | 90 (18.3) |  | 36 ( 25.0) | 2 ( 2.8) | 6 ( 16.7) | 3.E-04 |
|  | Travel Domestically (Between States) |  | 91 (18.5) |  | 31 ( 21.5) | 9 ( 12.7) | 7 ( 19.4) | 0.292 |
|  | Go to a Bar/Restaurant |  | 223 (45.2) |  | 87 ( 60.4) | 20 ( 28.2) | 15 ( 41.7) | 3.E-05 |
|  | Travel Internationally |  | 21 ( 4.3) |  | 6 ( 4.2) | 0 ( 0.0) | 2 ( 5.6) | 0.179 |
| Are you currently working as an essential employee? (NOT from home) If so, please select from the options below: | |  |  |  |  |  |  |  |
|  | No - I am not currently working as an essential employee |  | 6525 (80.1) |  | 105 ( 72.9) | 35 ( 45.5) | 25 ( 69.4) | 2.E-04 |
|  | Grocery/convenience store employee |  | 58 ( 0.7) |  | 0 ( 0.0) | 2 ( 2.6) | 1 ( 2.8) | 0.144 |
|  | Warehouse/factory worker |  | 98 ( 1.2) |  | 2 ( 1.4) | 2 ( 2.6) | 0 ( 0.0) | 0.565 |
|  | First responder (EMT, Firefighter, police, military) |  | 49 ( 0.6) |  | 0 ( 0.0) | 3 ( 3.9) | 0 ( 0.0) | 0.029 |
|  | Medical professional/staff member (doctor, nurse, clerk, janitorial staff, etc.) |  | 679 ( 8.3) |  | 18 ( 12.5) | 22 ( 28.6) | 6 ( 16.7) | 0.012 |
|  | Truck driver/delivery |  | 29 ( 0.4) |  | 1 ( 0.7) | 0 ( 0.0) | 0 ( 0.0) | 0.674 |
|  | Bus driver/train operator |  | 7 ( 0.1) |  | 1 ( 0.7) | 0 ( 0.0) | 0 ( 0.0) | 0.674 |
|  | Restaurant worker |  | 51 ( 0.6) |  | 0 ( 0.0) | 2 ( 2.6) | 0 ( 0.0) | 0.095 |
|  | Sanitation worker |  | 12 ( 0.1) |  | 0 ( 0.0) | 1 ( 1.3) | 0 ( 0.0) | 0.309 |
| Please select all the immune system conditions that apply to you. | |  |  |  |  |  |  |  |
|  | I have none of these conditions |  | 5635 (69.0) |  | 91 ( 62.8) | 50 ( 64.9) | 26 ( 72.2) | 0.567 |
|  | Type II Diabetes (high blood sugar) |  | 967 (11.8) |  | 20 ( 13.8) | 15 ( 19.5) | 2 ( 5.6) | 0.139 |
|  | Immunocompromised status |  | 697 ( 8.5) |  | 12 ( 8.3) | 5 ( 6.5) | 5 ( 13.9) | 0.418 |
|  | Autoimmune or rheumatologic disease |  | 1004 (12.3) |  | 25 ( 17.2) | 10 ( 13.0) | 8 ( 22.2) | 0.453 |
|  | Organ transplant |  | 165 ( 2.0) |  | 2 ( 1.4) | 2 ( 2.6) | 0 ( 0.0) | 0.563 |
|  | Type I Diabetes (high blood sugar) |  | 255 ( 3.1) |  | 6 ( 4.1) | 1 ( 1.3) | 0 ( 0.0) | 0.259 |
|  | HIV |  | 30 ( 0.4) |  | 0 ( 0.0) | 1 ( 1.3) | 0 ( 0.0) | 0.307 |
|  | Bone marrow transplant |  | 19 ( 0.2) |  | 0 ( 0.0) | 0 ( 0.0) | 0 ( 0.0) |  |
| Please select all respiratory conditions that apply to you. | |  |  |  |  |  |  |  |
|  | I have none of these conditions |  | 5215 (63.9) |  | 90 ( 62.1) | 41 ( 53.2) | 22 ( 61.1) | 0.432 |
|  | Sleep Apnea |  | 1809 (22.2) |  | 33 ( 22.8) | 20 ( 26.0) | 6 ( 16.7) | 0.547 |
|  | I use a home CPAP |  | 1432 (17.5) |  | 25 ( 17.2) | 18 ( 23.4) | 4 ( 11.1) | 0.261 |
|  | Asthma |  | 1195 (14.6) |  | 26 ( 17.9) | 13 ( 16.9) | 10 ( 27.8) | 0.344 |
|  | Chronic Obstructive Pulmonary Disease (COPD) |  | 312 ( 3.8) |  | 3 ( 2.1) | 6 ( 7.8) | 1 ( 2.8) | 0.102 |
|  | Emphysema |  | 94 ( 1.2) |  | 0 ( 0.0) | 4 ( 5.2) | 0 ( 0.0) | 0.008 |
|  | Cystic Fibrosis |  | 6 ( 0.1) |  | 0 ( 0.0) | 0 ( 0.0) | 1 ( 2.8) | 0.045 |
| Please select all the genitourinary/metabolic conditions that apply to you. | |  |  |  |  |  |  |  |
|  | I have none of these conditions |  | 7318 (89.6) |  | 132 ( 91.0) | 71 ( 92.2) | 31 ( 86.1) | 0.570 |
|  | Chronic Kidney Disease |  | 525 ( 6.4) |  | 8 ( 5.5) | 5 ( 6.5) | 1 ( 2.8) | 0.717 |
|  | Liver Disease |  | 200 ( 2.4) |  | 1 ( 0.7) | 2 ( 2.6) | 4 ( 11.1) | 0.003 |
|  | Gallbladder Disease |  | 134 ( 1.6) |  | 4 ( 2.8) | 0 ( 0.0) | 0 ( 0.0) | 0.205 |
|  | Pancreas Disease |  | 76 ( 0.9) |  | 1 ( 0.7) | 0 ( 0.0) | 0 ( 0.0) | 0.676 |
| Please select all the cardiovascular conditions that apply to you. | |  |  |  |  |  |  |  |
|  | I have none of these conditions |  | 4666 (57.1) |  | 90 ( 62.1) | 47 ( 61.0) | 27 ( 75.0) | 0.303 |
|  | Stroke |  | 219 ( 2.7) |  | 0 ( 0.0) | 1 ( 1.3) | 3 ( 8.3) | 0.001 |
|  | Hypertension (high blood pressure) |  | 2654 (32.5) |  | 40 ( 27.6) | 22 ( 28.6) | 5 ( 13.9) | 0.202 |
|  | Balloon angioplasty or percutaneuous coronary intervention |  | 302 ( 3.7) |  | 3 ( 2.1) | 1 ( 1.3) | 0 ( 0.0) | 0.652 |
|  | Arrythmias |  | 655 ( 8.0) |  | 15 ( 10.3) | 8 ( 10.4) | 1 ( 2.8) | 0.348 |
|  | Coronary artery bypass |  | 230 ( 2.8) |  | 2 ( 1.4) | 1 ( 1.3) | 0 ( 0.0) | 0.781 |
|  | Myocardial infarction |  | 213 ( 2.6) |  | 3 ( 2.1) | 2 ( 2.6) | 0 ( 0.0) | 0.637 |
|  | Congestive heart failure |  | 305 ( 3.7) |  | 2 ( 1.4) | 6 ( 7.8) | 1 ( 2.8) | 0.045 |
|  | Peripheral vascular disease |  | 174 ( 2.1) |  | 1 ( 0.7) | 2 ( 2.6) | 0 ( 0.0) | 0.353 |
|  | Blood clot or clotting disorder |  | 335 ( 4.1) |  | 4 ( 2.8) | 3 ( 3.9) | 3 ( 8.3) | 0.300 |
| Please select all the neurological conditions that apply to you. | |  |  |  |  |  |  |  |
|  | I have neither of these conditions |  | 7893 (96.6) |  | 140 ( 96.6) | 72 ( 93.5) | 36 (100.0) | 0.230 |
|  | Neurological disease |  | 259 ( 3.2) |  | 5 ( 3.4) | 3 ( 3.9) | 0 ( 0.0) | 0.503 |
|  | Dementia |  | 27 ( 0.3) |  | 0 ( 0.0) | 2 ( 2.6) | 0 ( 0.0) | 0.094 |
| Please select all the conditions/treatments that apply to you. | |  |  |  |  |  |  |  |
|  | I have none of these conditions |  | 7086 (86.8) |  | 129 ( 89.0) | 71 ( 92.2) | 32 ( 88.9) | 0.729 |
|  | Malignant solid tumor |  | 382 ( 4.7) |  | 4 ( 2.8) | 0 ( 0.0) | 4 ( 11.1) | 0.006 |
|  | Chemotherapy |  | 523 ( 6.4) |  | 7 ( 4.8) | 4 ( 5.2) | 1 ( 2.8) | 0.841 |
|  | Radiation Therapy |  | 650 ( 8.0) |  | 9 ( 6.2) | 3 ( 3.9) | 2 ( 5.6) | 0.769 |
|  | Lymphoma |  | 101 ( 1.2) |  | 2 ( 1.4) | 0 ( 0.0) | 0 ( 0.0) | 0.456 |
|  | Leukemia |  | 53 ( 0.6) |  | 2 ( 1.4) | 0 ( 0.0) | 0 ( 0.0) | 0.456 |
