# Supplementary material for: Exposure and risk factors for COVID-19 and the impact of staying home on Michigan residents
Source: PLoS One. 2021 Feb 8;16(2):e0246447. doi: 10.1371/journal.pone.0246447 (PMC7870003; doi:10.1371/journal.pone.0246447)
Supplement: S3 Table — (DOCX) [file pone.0246447.s003.docx]

| Table S3. Descriptive Characteristics of the COVID-19 Tested/Diagnosed Central Biorepository and COVID-19 Survey Participants | | | | | | | | |
| --- | --- | --- | --- | --- | --- | --- | --- | --- |
|  | **CBR Participants  (n = 76,358)** | | **CBR Participants Emailed**  **(n = 50,820)** | | | **COVID-19 Survey (n = 8,041)** | | |
| Variable | **Tested (n = 3,579)** | **Positive (n = 200)** | **Overall (n = 50,820)** | **Tested (n = 3050)** | **Positive (n = 160)** | **Overall (n = 8,041)** | **Tested (n = 927)** | **Positive (n = 132)** |
| Age Group, No.(%) |  |  |  |  |  |  |  |  |
| [0,18) | 0 (0) | 0 (0) | 0 (0) | 0 (0) | 0 (0) | 0 (0) | 0 (0) | 0 (0) |
| [18,35) | 372 (10.39) | 28 (14) | 6645 (13.08) | 343 (11.25) | 20 (12.5) | 715 (8.89) | 93 (10.03) | 24 (18.18) |
| [35,50) | 662 (18.50) | 50 (25) | 10467 (20.60) | 605 (19.84) | 45 (28.13) | 1379 (17.15) | 186 (20.06) | 39 (29.55) |
| [50,65) | 1158 (32.36) | 66 (33) | 16665 (32.79) | 991 (32.49) | 49 (30.63) | 2740 (34.08) | 325 (35.06) | 45 (34.09) |
| ≥ 65 | 1387 (38.75) | 56 (28) | 17043 (33.54) | 1111 (36.43) | 46 (28.75) | 3207 (39.88)) | 323 (34.84) | 24 (18.18) |
| Male Sex (%) | 1595 (44.57) | 89 (44.5) | 22353 (43.98) | 1340 (43.93) | 68 (42.5) | 3310 (41.53) | 341 (37.15) | 52 (39.69) |
| Race Ethnicity, No.(%) |  |  |  |  |  |  |  |  |
| NHEA | 2846 (79.52) | 119 (59.5) | 43639 (85.87) | 2540 (83.28) | 98 (61.25) | 7387 (91.87) | 818 (88.24) | 108 (81.81) |
| NHAA | 358 (10.00) | 59 (29.5) | 2698 (5.31) | 265 (8.69) | 50 (31.25) | 233 (2.90) | 47 (5.07) | 13 (9.85) |
| Other Ethnicity | 70 (1.96) | 2 (1) | 1047 (2.06) | 66 (2.16) | 2 (1.25) | 325 (4.04) | 49 (5.29) | 10 (7.58) |
| Unknown Ethnicity | 87 (2.43) | 4 (2) | 1592 (3.13) | 72 (2.36) | 1 (0.63) | 96 (1.19) | 13 (1.40) | 1 (0.77) |
| Abbreviations: Non-Hispanic European American, NHEA; Non-Hispanic African American, NHAA | | | | | | | | |
