# Supplementary material for: Exposure and risk factors for COVID-19 and the impact of staying home on Michigan residents
Source: PLoS One. 2021 Feb 8;16(2):e0246447. doi: 10.1371/journal.pone.0246447 (PMC7870003; doi:10.1371/journal.pone.0246447)
Supplement: S4 Table — (DOCX) [file pone.0246447.s004.docx]

| **Table S4. Michigan Medicine Precision Health COVID-19 Survey.** Skip logic was applied, meaning most participants only answered 50% of the 96 total questions. | | | | | |
| --- | --- | --- | --- | --- | --- |
|  | | | | | |
| **Subject** |  | **Question** |  | **Answer type** |  |
| **COVID-19 Symptoms, Exposure, Diagnosis, Severity** |  | 1.Were you diagnosed with COVID-19? |  | Yes, diagnosed with Test  Yes, diagnosed by a physician without a test  Yes, self-diagnosed due to symptoms  No, but I was exposed to someone who was diagnosed with COVID-19  No, but I was exposed to someone suspected of having COVID-19  No |  |
|  |  | If 1a = “Yes diagnosed with test”  1a1. Where were you tested |  | Hospital (Including Emergency Room)  Drive-thru  Clinic  Urgent Care |  |
|  |  | If 1a “Yes diagnosed with” test is not selected  1a2. Were you tested for COVID-19 at any point in time, if so where? |  | I was not tested  Hospital (Including Emergency Room)  Drive thru  Clinic  Urgent care |  |
|  |  | If 1a2: “I was not tested” is not selected  1a3. When were you tested?  1a3. Was your test for COVID-19 positive or negative |  | MM/DD/YYY  Positive  Negative  Results Pending |  |
|  |  | If 1a = “No – But I was exposed to someone who was diagnosed with COVID-19”\ “No – But I was exposed to someone suspected of having COVID-19”  1a4. How were you exposed to COVID-19/Suspected COVID-19 |  | Travel Related  Family member was sick  Friend or other social contact was sick  Coworker was sick  Work in high risk environment (first responder/hospital employee)  Work in a service job (grocery store employee, sanitation worker, warehouse workers, etc.)  Unsure  Other |  |
|  |  | 2. What COVID-19 Symptoms did you have (Check all that apply) |  | Fever, Cough, Runny Nose, Sore Throat, Sneezing, Congestion, Loss of Smell/Taste, Nausea/Diarrhea, Shortness of Breath, Headache, Fatigue, Chest Pain, Muscle Aches, Other, No Symptoms |  |
|  |  | 2a. If “Shortness of Breath” Is selected  2a1. Please indicate if you would have shortness of breath due to the following tasks (at the peak of your symptoms) |  | At rest, doing the dishes, showering/bathing, walking, vacuuming and cleaning, walking up the stairs |  |
|  |  | 3. When did you begin having symptoms? |  | MM/DD/YYY |  |
|  |  | 4. Approximately how many total days did your symptoms last? |  | #days  Still having symptoms |  |
|  |  | If 1a=yes, or 1a3= positive  5. Were you admitted to the hospital due to COVID-19 symptoms |  | Yes/No |  |
|  |  | If 5 = yes  5a. Are you still in the hospital?  5b. How many days were/have you been in the hospital?  5c. Were you on a ventilator at some point during your stay in the hospital  5d. While you were in the hospital for COVID-19, did you have any heart complications (please select all that apply) |  | Yes/No  #Days  Yes/No  Congestive Heart Failure/Cardiomyopathy, Heart Disease, Heart Attack, Irregular Heart Rhythm (AFib), ECG/EKG Abnormalities, Angina (Chest Pain), Other, No, Not sure |  |
|  |  | If 1a=yes or 1a3 = positive  6. When you were diagnosed with COVID-19 were you also tested for having the flu (influenza) |  | Yes/No |  |
|  |  | If in 2 “No symptoms” is not selected  7a. In the two weeks prior to you showing symptoms, did you have exposure to a person diagnosed with COVID-19?  7b. Which of the following things did you do in the two weeks prior to showing symptoms?  7c. Have you been around family in the two weeks prior to showing symptoms?  If 7c = yes  7d. Have any of your family members been diagnosed with COVID-19 within two weeks after you saw them?  7e. Have you been around anyone outside your household in the two weeks prior to showing symptoms?  If 7e = yes  7f. Has anyone outside your household been diagnosed with COVID-19 within two weeks after you saw them last? |  | Yes/No/Unsure  Travel Internationally, Travel Domestically (between states), Travel within your state, Go to the grocery store, Go to a Bar/Restaurant, Participate in any Festivals/Events of over 50 people, Work in direct contact with the Public (E.g. grocery store employee, cashier, clerk etc.), None of these things  Yes/No  Yes/No/Unsure  Yes/No  Yes/No/Unsure |  |
|  |  | 8a.Not including yourself how many people in EACH age Group currently reside in your household.  8b. If applicable, please indicate how many have been diagnosed with COVID-19 |  | # People  #People Diagnosed |  |
|  |  | 9. Is there an individual in your household (not including yourself) that is considered “high risk” for COVID-19? |  | Yes/No |  |
|  |  | If in 2 “No Symptoms” is not selected  10. Have you been around anyone outside your household in the two weeks prior to showing symptoms?  10b. Has anyone outside your household been diagnosed with COVID-19 within two weeks after you saw them last? |  | Yes/No  Yes/No/Unsure |  |
|  |  | 11a. Are you currently working as an essential employee (NOT from home)? If so please select from the options below.  If 11a “No I am not currently working as an essential employee” is NOT selected”  11a1. Approximately how many hours per week are you CURRENTLY working?  If 11a “Medical Professional/Staff Member is selected”  11b1. What type of medical professional or supporting staff member are you?  11b2. As a medical professional/staff member have you been in direct contact with patients who have either had COVID-19 or were exposed to it?  11b3. Is there a medical professional/clinical staff member currently living in your household (NOT including yourself)  If 11b3 “No” is NOT selected  11b4. Has this medical professional/staff member been in direct contact with patients who have either had COVID-19 or were exposed to it?  If 11a “First Responder” is selected  11c1. What type of first responder are you?  11c2. As a first responder have you been in direct contact with patients who have either had COVID-19 or were exposed to it?  11c3. Is there a medical professional/clinical staff member currently living in your household? (Not including yourself)  In 11c3. If “No” is NOT selected  11c4. Has this medical professional/staff member been in direct contact with patients who have either had COVID-19 or were exposed to it? |  | Medical Professional/staff member (doctor, nurse, clerk, janitorial staff, etc.), First Responder (EMT, Firefighter, police, military), Grocery/Convenience store employee, Warehouse/Factory Worker, Sanitation Worker, Truck driver/delivery, Bus driver/Train operator, Restaurant Worker, Other  No- I am not currently working as an essential employee  0-10, 11-20, 21-30, 31-40, 41-50, 51-60, 61-70, 71+  Physician, Nurse, Other medical professional (e.g. MA, NA, Technician), other clinical supporting staff (e.g. clerk, janitorial staff, research)  Yes/No/Unsure  No, Physician, Nurse, Other medical professional (e.g. MA, NA, Technician), Other Clinical supporting staff member (e.g. clerk, janitorial staff, research)  Yes/No/Unsure  Police Officer, Firefighter, Emergency Medical Technician (EMT/Paramedic), Military (National Guard), Other  Yes/No/Unsure  No, Physician, Nurse, Other medical professional (e.g. MA, NA, Techician), Other clinical supporting staff member (e.g. clerk, janitorial staff, research)  Yes/No/Unsure |  |
|  |  | 12. How many times have you gotten the flu (influenza) in the past year? (Fever of 100.4 or higher). |  | 0,1-2,3-4, 5+ |  |
|  |  | 13. Did you get a flu shot in the past year? |  | Yes/No |  |
|  |  | 14. How many times have you gotten the common cold in the past year? |  | 0,1-2,3-4,5+ |  |
|  |  | 15. Have you ever been hospitalized for a viral infection (NOT relating to COVID-19)? |  | Yes/No |  |
|  |  | If 1a = yes or 1a3 = positive  16a. Were you admitted to the hospital due to COVID-19 symptoms?  If 16a = Yes  16b. Are you still in the hospital?  16c. How many days were/have you been hospitalized?  16d. Were you on a ventilator at some point during your stay in the hospital?  16e. While you were in the hospital for COVID-19, did you have any heart complications (Please select all that apply) |  | Yes/No  Yes/No  #days  Yes/No  Congestive Heart Failure, Cardiomyopathy/Heart Disease, Heart Attack, Irregular Heart Rhythm (Afib), ECG/EKG Abnormalities, Angina (Chest Pain), Other, No, Not sure |  |
|  |  |  |  |  |  |
| **COVID-19 Concerns** |  | 1. How concerned have you been about the novel COVID-19 Pandemic in the past 7 days? |  | Not Concerned = 0, Extremely Concerned = 10 |  |
|  |  | 2. How concerned are you about the following with respect to COVID-19?  2a. If “Not diagnosed with COVID-19”/”Exposed to someone who was diagnosed/suspected to have COVID-19” is selected  2a1. Contracting COVID-19  2a2. Someone close to you contracting COVID-19  2a3. Getting into serious financial trouble  2a4. Losing your job  2a5. That it will be a long time before your life returns to normal  2a6. Not seeing friends and family  2b. If “Yes” Diagnosed with COVID-19/ Diagnosed by physician without a test/Self-Diagnosed due to symptoms is selected  2b1. Someone close to you contracting COVID-19  2b2. Getting into serious financial trouble  2b3. Losing your job  2b4. That it will be a long time before your life returns to normal  2b5. Not seeing friends and family |  | Not Concerned, Slightly Concerned, Very Concerned, Extremely Concerned  Not Concerned, Slightly Concerned, Very Concerned, Extremely Concerned  Not Concerned, Slightly Concerned, Very Concerned, Extremely Concerned  Not Concerned, Slightly Concerned, Very Concerned, Extremely Concerned  Not Concerned, Slightly Concerned, Very Concerned, Extremely Concerned  Not Concerned, Slightly Concerned, Very Concerned, Extremely Concerned  Not Concerned, Slightly Concerned, Very Concerned, Extremely Concerned  Not Concerned, Slightly Concerned, Very Concerned, Extremely Concerned  Not Concerned, Slightly Concerned, Very Concerned, Extremely Concerned  Not Concerned, Slightly Concerned, Very Concerned, Extremely Concerned  Not Concerned, Slightly Concerned, Very Concerned, Extremely Concerned |  |
|  |  | 3. What precautions are you taking to protect yourself or others from COVID-19 (check all that apply) |  | Frequent hand washing, hand disinfectant use, cover face while sneezing/coughing, wearing a mask, avoiding public transport, avoid travel in general, work from home, social distancing (but not from other members of the household), self-isolation, other, none of these |  |
|  |  | 4. Where have you been getting your information/advice regarding COVID-19 in the last few days? |  | Medical Professionals, Government Health Websites (WHO, CDC, NIH), Other health websites (WedMD, MayoClinic), National News (CNN, FOX, NBC), Print News (NYTimes, Washington Post, WSJ, Local Paper), Social Media (Facebook, Instagram, Twitter), Friends/Family, Other |  |
|  |  | 5. Rate how much you agree with the following statement: Since the outbreak of COVID-19 Pandemic, I see others in my community as a threat to my well-being |  | Totally Disagree, Slightly Disagree, Neutral, Slightly Agree, Totally Agree |  |
|  |  | 6. Rate how much you agree with the following statement: Since the outbreak of COVID-19 Pandemic, my thoughts have become more fearful/negative towards Asian-American citizens |  | Totally Disagree, Slightly Disagree, Neutral, Slightly Agree, Totally Agree |  |
|  |  | 7. Rate how much you agree with the following statement: Since the outbreak of COVID-19 Pandemic, I have witnessed racial prejudice/ discrimination against Asian-Americans |  | Totally Disagree, Slightly Disagree, Neutral, Slightly Agree, Totally Agree |  |
|  |  | 8a. Have you had any blood-relatives diagnosed with COVID-19? If so, please select from the options below and state how many people  8b. If in 8a any blood relatives were selected as diagnosed with COVID-19?  8b1. Of your blood relatives that were diagnosed with COVID-19, have any passed away from COVID-19? If so, please select from the options below and state how many people |  | No- No blood relatives of mine have been diagnosed with COVID-19, unsure, Grandmother (Paternal or Maternal), Grandfather (Paternal or Maternal), Mother, Father, Sister, Brother, Daughter, Son, Granddaughter, Grandson  No- No blood relatives of mine have been diagnosed with COVID-19, unsure, Grandmother (Paternal or Maternal), Grandfather (Paternal or Maternal), Mother, Father, Sister, Brother, Daughter, Son, Granddaughter, Grandson |  |
| **Participant Characteristics** |  | 1. What is your date of birth? |  | MM/DD/YYYY |  |
|  |  | 2. What is your sex?  2a. If “Female” is selected  2a1. Are you pregnant  2a2. When are you due?  3. What is your current height and weight? |  | Male/Female/Other/Prefer not to answer  Yes/No/Not sure  MM/YYYY  Feet, Inches, Weight (pounds) |  |
|  |  | 4. What is your race? (check all that apply) |  | American Indian or Alaskan Native, Asian, Black or African American, Native Hawaiian or Pacific Islander, White/Caucasian, Unknown, Other, Prefer not to answer. |  |
|  |  | 5. What is your ethnicity |  | Hispanic or Latino, Non-Hispanic or Latino, Unknown, Other, Prefer not to answer |  |
|  |  | 6. What is the zip code of where you are currently staying? |  | Numerical Zip Code |  |
|  |  | 7. What is your average annual household income from all sources? |  | Less than $10,000, $10,000-$40,000, $40,000-$60,000, $60,000-$80,000, $80,000-$100,000, $100,000-$200,000, $200,000 or more |  |
|  |  | 8. What is the highest grade or year of school that you have completed? |  | Never attended school, Primary School (Grades 1-4), Middle School (Grades 5-8), Some high school (Grades 9-11), High school graduate (Grade 12 or G.E.D), Associate Degree (2 or more years of college), Bachelors Degree (4 or more years of college), Advanced Degree (Masters, Doctorate) |  |
|  |  | 9. How many cars/automobiles do you have in your household? |  | 0,1,2,3,4,5,6,7,8,9,10+ |  |
|  |  | 10. What is your primary mode of transportation? |  | Car/Automobile, Bus/Public Transportation, Bicycle, Motorcycle/Motor Scooter, Walk, Other |  |
|  |  | 11. What is your current living situation |  | I own a house, A family member owns the house I live in, I rent a house/apartment, other |  |
|  |  |  |  |  |  |
| **Mental Health** |  | 1. Using the following scale, indicate for each item your severity OVER THE PAST WEEK by picking the appropriate choice:  1a. Fatigue  1b. Trouble thinking or remembering  1c. Waking up tired (unrefreshed)  1d. If “No problem” was NOT selected in 1a,1b,1c,  1d1. Have your problems with these symptoms been present for 3 or more months? |  | No Problem, Mild, Moderate, Severe  No Problem, Mild, Moderate, Severe  No Problem, Mild, Moderate, Severe  Yes/No |  |
|  |  | 2. During the past 6 MONTHS have you had any of the following symptoms?  2a. Pain or cramps in the lower abdomen?  2b. Depression  2c. Headache |  | Yes/ No  Yes/No  Yes/No |  |
|  |  | 3. Please respond to each item by picking the appropriate choice  3a. Are you able to do chores such as vacuuming or yard work?  3b. Are you able to go up and down stairs at a normal pace?  3c. Are you able to go for a walk of at least 15 minutes?  3d. Are you able to run errands and shop? |  | Without any Difficulty, With a little difficulty, With some difficulty, With much difficulty, Unable to do  Without any Difficulty, With a little difficulty, With some difficulty, With much difficulty, Unable to do  Without any Difficulty, With a little difficulty, With some difficulty, With much difficulty, Unable to do  Without any Difficulty, With a little difficulty, With some difficulty, With much difficulty, Unable to do |  |
|  |  | 4. In the last 7 days…  4a. I felt worthless  4b. I felt hopeless  4c. I felt depressed  4d. I felt hopeless |  | Never, Rarely, Sometimes, Often, Always  Never, Rarely, Sometimes, Often, Always  Never, Rarely, Sometimes, Often, Always  Never, Rarely, Sometimes, Often, Always |  |
|  |  | 5. In the past 7 days…  5a. I felt fearful  5b. I felt it hard to focus on anything other than my anxiety  5c. My worries overwhelmed me  5d. I felt uneasy |  | Never, Rarely, Sometimes, Often, Always  Never, Rarely, Sometimes, Often, Always  Never, Rarely, Sometimes, Often, Always  Never, Rarely, Sometimes, Often, Always |  |
|  |  | 6. In the past 7 days, my sleep quality was |  | Very poor, poor, fair, good, very good |  |
|  |  | 7. Please respond to each item by picking the appropriate choice  7a. Do you have someone to help you if you are confined in bed?  7b. Do you have someone to take you to the doctor if you need it?  7c. Do you have someone to run errands if you need it? |  | Never, Rarely, Sometimes, Often, Always  Never, Rarely, Sometimes, Often, Always  Never, Rarely, Sometimes, Often, Always |  |
| **Medical/ Family History** |  | 1. Do you have a Primary Care Physician (PCP/Family Doctor)  1a. If “yes” is selected  1a1. Approximately how often do you see your Primary Care Physician (PCP/Family Doctor)  1a2. Is your Primary Care Physician (PCP/Family Doctor) a University of Michigan doctor? |  | Yes/ No/ Unsure  Less than once a year, Once a year, More than once a year  Yes/No/ Unsure |  |
|  |  | 2. What is your blood type? |  | Not sure, A+, A-, B+, B-, AB+, AB-, O+, O- |  |
|  |  | 3. Please select all the immune system conditions that apply to you |  | HIV  Immunocompromised status  Organ transplant  Bone Marrow transplant  Autoimmune or rheumatologic disease (e.g. Rheumatoid arthritis, systemic lupus erythematosus, multiple sclerosis, inflammatory bowel disease)  Type I Diabetes (High blood sugar)  Type II Diabetes (High blood sugar)  I have none of these conditions |  |
|  |  | 4. Please select all respiratory conditions that apply to you |  | Asthma  Chronic Obstructive Pulmonary Disease (COPD)  Cystic Fibrosis  Emphysema  Sleep Apnea  I use a home CPAP  I have none of these conditions |  |
|  |  | 5. Please select all genitourinary/metabolic conditions that apply to you |  | Chronic Kidney Disease  Liver Disease  Gallbladder Disease  Pancreas Disease  I have none of these conditions |  |
|  |  | 6. Please select all cardiovascular conditions that apply to you |  | Balloon Angioplasty or percutaneous coronary intervention  Coronary Artery Bypass  Congestive Heart Failure  Hypertension (high blood pressure)  Myocardial Infarction  Peripheral Vascular Disease  Blood clot or clotting disorder  Stroke  Arrhythmias  I have none of these conditions |  |
|  |  | 7. Please select all the neurological conditions that apply to you |  | Dementia  Neurological Disease  I have neither of these conditions |  |
|  |  | 8. Please select all conditions/treatments that apply to you |  | Leukemia  Lymphoma  Malignant solid tumor  Chemotherapy  Radiation therapy  I have none of these conditions |  |
|  |  | 9. Please select all conditions that apply to your family members (Mother, Father, Grandmother (Paternal/Maternal), Grandfather (Paternal/Maternal), Sister, Brother, Daughter, Son  9a. Deceased  9b. Cancer  9c. Chronic Kidney Disease  9d. COPD (Lung Disease)  9e. Coronary Artery Disease  9f. Crohn’s Disease  9g. Depression/ Anxiety  9h. Heart Attack  9i. High/ Low Cholesterol  9j. Hypertension (High Blood Pressure)  9k. Liver Disease  9l. Asthma  9m. Stroke  9n. Diabetes (High Blood Pressure)  9o. Heart Disease  9p. Irregular Heart Rhythm (Afib)  9q. Autoimmune Diseases (HIV, AIDS, MS, Lupus, etc.)  9r. Any organ transplant |  | Yes = selected / No = Unselected  Yes = selected / No = Unselected  Yes = selected / No = Unselected  Yes = selected / No = Unselected  Yes = selected / No = Unselected  Yes = selected / No = Unselected  Yes = selected / No = Unselected  Yes = selected / No = Unselected  Yes = selected / No = Unselected  Yes = selected / No = Unselected  Yes = selected / No = Unselected  Yes = selected / No = Unselected  Yes = selected / No = Unselected  Yes = selected / No = Unselected  Yes = selected / No = Unselected  Yes = selected / No = Unselected  Yes = selected / No = Unselected  Yes = selected / No = Unselected |  |
| **Health Behaviors** |  | 1. During the “Stay Home, Stay Safe” social distancing, please indicate how your behaviors have changed relative to your usual behavior before social distancing  1a. I have increased moderate to strenuous exercise  1b. I have increased my alcohol consumption  1c. I have increased my drug use  1d. I have increased my tobacco use  1e. I have improved my sleep habits  1f. I have improved my nutrition (Dietary Habits)  1g. I have gained weight |  | Strongly Disagree, Disagree, About the same, Agree, Strongly Agree, Not Applicable  Strongly Disagree, Disagree, About the same, Agree, Strongly Agree, Not Applicable  Strongly Disagree, Disagree, About the same, Agree, Strongly Agree, Not Applicable  Strongly Disagree, Disagree, About the same, Agree, Strongly Agree, Not Applicable  Strongly Disagree, Disagree, About the same, Agree, Strongly Agree, Not Applicable  Strongly Disagree, Disagree, About the same, Agree, Strongly Agree, Not Applicable  Strongly Disagree, Disagree, About the same, Agree, Strongly Agree, Not Applicable |  |
|  |  | 2. Please indicate your physical activity participation in relation to your states shelter at home recommendations. In an average week  How many DAYS do you do a total of 30 minutes or more of physical activity, which is enough to raise your breathing rate?  This may include sport, exercise, and brisk walking or cycling for recreation or to get to and from places, but should not include housework or physical activity that may be a part of your job.  2a. Before COVID shelter at home recommendations  2b. After COVID shelter at home recommendations |  | 1,2,3,4,5,6,7  1,2,3,4,5,6,7 |  |
|  |  | 3. Do you use or have you used any tobacco/nicotine products?  3a. If “Current User” or “I have switched from one product to another is selected”  3a1. Please select all the tobacco/nicotine products that you currently use  3b. If “Current User” or “I have switched from one product to another” is selected  3b1. Please state how many years you have been using each product. (Select only the products that you currently use)  3b1a. Cigarettes  3b1b. Cigars or Pipes  3b1c. Hand Rolled Cigarettes  3b1d. Vape Pen/ E-Cigarettes  3b1e. Smokeless tobacco  3b1f. Other  3c. If “Current User” or “I have switched from one product to another is selected”  3c1. Please state how many years you have been using each product. (Please select only the products that you no longer use)  3c1a. Cigarettes  3c1b. Cigars or Pipes  3c1c. Hand Rolled Cigarettes  3c1d. Vape Pen/ E-Cigarettes  3c1e. Smokeless tobacco  3c1f. Other  3d. If “Never Used” is NOT selected  3d1. On average, how many cigarettes (or nicotine equivalent) do/did you smoke per day? (one pack is approximately 20 cigarettes). |  | Current User, Former User, Never Used, I have switched from one product to another  Cigarettes, Cigars or pipes, Hand Rolled Cigarettes, Vape pen/ E-Cigarettes, Smokeless tobacco, Other  # Years Used  # Years Used  # Years Used  # Years Used  # Years Used  # Years Used  # Years since used  # Years since used  # Years since used  # Years since used  # Years since used  # Years since used  # Cigarettes/Day |  |
|  |  | 4. During the “Stay Home, Stay Safe” social distancing please indicate how your behaviors have changed relative to your usual behavior before social distancing  4a. I have increased my opioid pain medication use (e.g. Vicodin, Norco, Oxycodone, Morphine)  4a1. If “Never Used” is NOT selected  4a1a. Was this opioid ____?  4a12. Approximately how many of the last 30 days have you used an opioid?  4b. I have increased my benzodiazepine use (e.g. Xanax, Valium)  4b1. If “Never Used” is NOT selected  4b1a. Was this Benzodiazepine ____?  4b1b. Approximately how many of the last 30 days have you used a benzodiazepine?  4c. I have increased my marijuana/cannabis use  4c1. If “Never Used” is NOT selected  4c1a. What is the reason for which you are using the marijuana/cannabis (check all that apply) |  | Never Used, Strongly Disagree, Disagree, About the same, Agree, Strongly Agree  Prescribed to you recently, Old prescription/ left over medication, Someone else’s opioid, Prefer not to answer  # days  Never Used, Strongly Disagree, Disagree, About the same, Agree, Strongly Agree  Prescribed to you recently, Old prescription/ left over medication, Someone else’s opioid, Prefer not to answer  # days  Never Used, Strongly Disagree, Disagree, About the same, Agree, Strongly Agree  Chronic Pain, Migraine Headaches, Nausea, Glaucoma, Anxiety, Sleep Issues, Other |  |
|  |  | 5a. (If Male) In the PAST 12 MONTHS, how often have you had 5  or more drinks containing alcohol in one day? One standard drink  is about 1 small glass of wine (5oz), 1 beer (12oz), or 1 single shot  of liquor.  5a. (If Female) In the PAST 12 MONTHS, how often have you had 4  or more drinks containing alcohol in one day? One standard drink i  s about 1 small glass of wine (5oz), 1 beer (12oz), or 1 single shot  of liquor. |  | Daily or Almost Daily, Weekly, Monthly, Less than Monthly, Never  Daily or Almost Daily, Weekly, Monthly, Less than Monthly, Never |  |
|  |  | 6. Please consider overall body pain you may have. Use the  scale below, where 0 indicates “no pain” and 10 indicates  the “worst pain you could possibly imagine”  6a. Rate your pain at its worst in the last week (scale 0-10)    6b. Rate your pain on average in the last week (scale 0-10)    6c. If “0” is not selected on 6a or 6b  6c1. On the image below, CHECK ALL areas of your body  where you have felt persistent or recurrent pain present  for the last 3 months or longer (chronic pain) if you do not have  chronic pain check that. |  | 0,1,2,3,4,5,6,7,8,9,10  0,1,2,3,4,5,6,7,8,9, 10  No chronic pain or selected area associated with their pain |  |
