# Supplementary material for: Exposure and risk factors for COVID-19 and the impact of staying home on Michigan residents
Source: PLoS One. 2021 Feb 8;16(2):e0246447. doi: 10.1371/journal.pone.0246447 (PMC7870003; doi:10.1371/journal.pone.0246447)
Supplement: S5 Table — (DOCX) [file pone.0246447.s005.docx]

| **Table S5.** Lab-Confirmed COVID-19 Cases in Washtenaw County by Race (as of 7/16; in percentage) | | | | | |
| --- | --- | --- | --- | --- | --- |
|  |  | **cases** | **hospitalized** | **deaths** | **county population** |
| **Racial Group** | |  |  |  |  |
|  | Am. Indian or Alaska Native | <1% | 0% | 0% | 0.4% |
|  | Asian | 3% | 4% | 1% | 9.7% |
|  | Black or African American | 32% | 40% | 30% | 12.3% |
|  | Native Hawaiian or Pac. Islander | <1% | <1% | 2% | 0.1% |
|  | White | 59% | 54% | 67% | 74.1% |
|  | Unknown | 5% | 2% | 0% | - |
| **Ethnic Group** | |  |  |  |  |
|  | Hispanic or Latino (all races) | 4% | 3% | 2% | 4.8% |
