# Supplementary material for: Exposure and risk factors for COVID-19 and the impact of staying home on Michigan residents
Source: PLoS One. 2021 Feb 8;16(2):e0246447. doi: 10.1371/journal.pone.0246447 (PMC7870003; doi:10.1371/journal.pone.0246447)
Supplement: S6 Table — (DOCX) [file pone.0246447.s006.docx]

| **Table S6.** Demographic, social economic status, environmental factors, and self-reported health conditions by COVID status and severity | | | | | | | | | | | |
| --- | --- | --- | --- | --- | --- | --- | --- | --- | --- | --- | --- |
|  |  |  | **Overall** |  | **COVID status** | |  |  | **COVID+ severity** | |  |
|  |  |  |  |  | No | Yes | p |  | Mild-to-moderate | Severe | p |
|  |  | n | 8041 |  | 7909 | 132 |  |  | 102 | 30 |  |
| Race | |  |  |  |  |  |  |  |  |  |  |
|  | African American |  | 233 ( 3.1) |  | 220 ( 2.9) | 13 ( 10.7) | 5.E-06 |  | 6 ( 6.3) | 7 ( 26.9) | 0.005 |
|  | European American |  | 7387 (96.9) |  | 7279 (97.1) | 108 ( 89.3) |  |  | 89 ( 93.7) | 19 ( 73.1) |  |
| Sex | |  |  |  |  |  |  |  |  |  |  |
|  | Female |  | 4661 (58.5) |  | 4582 (58.4) | 79 ( 60.3) | 0.668 |  | 67 ( 66.3) | 12 ( 40.0) | 0.011 |
|  | Male |  | 3310 (41.5) |  | 3258 (41.6) | 52 ( 39.7) |  |  | 34 ( 33.7) | 18 ( 60.0) |  |
| Age | |  |  |  |  |  |  |  |  |  |  |
|  |  |  | 59.15 (14.79) |  | 59.28 (14.75) | 51.20 (14.94) | 1.E-09 |  | 49.44 (14.89) | 57.20 (13.73) | 0.014 |
| BMI | |  |  |  |  |  |  |  |  |  |  |
|  |  |  | 29.12 (6.64) |  | 29.11 (6.64) | 29.67 (6.95) | 0.341 |  | 29.56 (6.30) | 30.03 (8.85) | 0.747 |
| BMI category | |  |  |  |  |  |  |  |  |  |  |
|  | underweight |  | 73 ( 0.9) |  | 70 ( 0.9) | 3 ( 2.3) | 0.151 |  | 1 ( 1.0) | 2 ( 6.7) | 0.427 |
|  | normal |  | 2179 (28.2) |  | 2152 (28.4) | 27 ( 21.1) |  |  | 21 ( 21.4) | 6 ( 20.0) |  |
|  | overweight |  | 2582 (33.5) |  | 2538 (33.5) | 44 ( 34.4) |  |  | 35 ( 35.7) | 9 ( 30.0) |  |
|  | obese |  | 2881 (37.3) |  | 2827 (37.3) | 54 ( 42.2) |  |  | 41 ( 41.8) | 13 ( 43.3) |  |
| Education | |  |  |  |  |  |  |  |  |  |  |
|  | High school or less |  | 1365 (17.1) |  | 1340 (17.0) | 25 ( 19.2) | 0.830 |  | 15 ( 15.0) | 10 ( 33.3) | 0.176 |
|  | Associate degree |  | 1279 (16.0) |  | 1257 (16.0) | 22 ( 16.9) |  |  | 18 ( 18.0) | 4 ( 13.3) |  |
|  | Bachelor's degree |  | 2492 (31.2) |  | 2451 (31.2) | 41 ( 31.5) |  |  | 32 ( 32.0) | 9 ( 30.0) |  |
|  | Advanced degree |  | 2860 (35.8) |  | 2818 (35.8) | 42 ( 32.3) |  |  | 35 ( 35.0) | 7 ( 23.3) |  |
| Income | |  |  |  |  |  |  |  |  |  |  |
|  | <40 |  | 1070 (14.1) |  | 1055 (14.2) | 15 ( 11.5) | 0.259 |  | 10 ( 10.0) | 5 ( 16.7) | 0.038 |
|  | 40-100 |  | 3300 (43.6) |  | 3249 (43.7) | 51 ( 39.2) |  |  | 45 ( 45.0) | 6 ( 20.0) |  |
|  | >100 |  | 3201 (42.3) |  | 3137 (42.2) | 64 ( 49.2) |  |  | 45 ( 45.0) | 19 ( 63.3) |  |
| Current living situation | |  |  |  |  |  |  |  |  |  |  |
|  | A family member owns the house I live in |  | 465 ( 5.8) |  | 454 ( 5.8) | 11 ( 8.6) | 0.177 |  | 10 ( 10.0) | 1 ( 3.6) | 0.227 |
|  | I own a house |  | 6546 (81.9) |  | 6450 (82.0) | 96 ( 75.0) |  |  | 74 ( 74.0) | 22 ( 78.6) |  |
|  | I rent a house/apartment |  | 794 ( 9.9) |  | 779 ( 9.9) | 15 ( 11.7) |  |  | 13 ( 13.0) | 2 ( 7.1) |  |
|  | Other |  | 191 ( 2.4) |  | 185 ( 2.4) | 6 ( 4.7) |  |  | 3 ( 3.0) | 3 ( 10.7) |  |
| Have any of your family members been diagnosed with COVID-19 within two weeks after you saw them last? | |  |  |  |  |  |  |  |  |  |  |
|  | Yes |  | 44 (15.5) |  | 13 ( 6.8) | 31 ( 33.3) | 6.E-08 |  | 16 ( 24.2) | 15 ( 55.6) | 0.009 |
|  | No |  | 219 (77.1) |  | 160 (83.8) | 59 ( 63.4) |  |  | 47 ( 71.2) | 12 ( 44.4) |  |
|  | Unsure |  | 21 ( 7.4) |  | 18 ( 9.4) | 3 ( 3.2) |  |  | 3 ( 4.5) | 0 ( 0.0) |  |
| Has anyone outside of your household been diagnosed with COVID-19 within two weeks after you saw them last? | |  |  |  |  |  |  |  |  |  |  |
|  | Yes |  | 38 (13.5) |  | 19 ( 9.9) | 19 ( 21.1) | 0.040 |  | 13 ( 18.8) | 6 ( 28.6) | 0.567 |
|  | No |  | 150 (53.4) |  | 108 (56.5) | 42 ( 46.7) |  |  | 34 ( 49.3) | 8 ( 38.1) |  |
|  | Unsure |  | 93 (33.1) |  | 64 (33.5) | 29 ( 32.2) |  |  | 22 ( 31.9) | 7 ( 33.3) |  |
| Approximately how many days did your symptoms last? (time from symptom onset to completely recovered) | |  |  |  |  |  |  |  |  |  |  |
|  |  |  | 14.22 (10.90) |  | 11.89 (10.12) | 18.66 (11.01) | 8.E-08 |  | 17.47 (10.61) | 22.40 (11.59) | 0.035 |
| Please indicate your physical activity participation in relation to shelter at home recommendations. | |  |  |  |  |  |  |  |  |  |  |
|  | Before |  | 3.68 (1.85) |  | 3.68 (1.85) | 3.72 (1.85) | 0.836 |  | 3.67 (1.84) | 3.90 (1.94) | 0.625 |
|  | After |  | 3.74 (2.05) |  | 3.75 (2.05) | 2.94 (1.99) | 2.E-04 |  | 3.06 (2.07) | 2.35 (1.50) | 0.186 |
| What precautions are you taking to protect yourself or others from COVID-19? | |  |  |  |  |  |  |  |  |  |  |
|  | Frequent hand washing |  | 7725 (96.1) |  | 7600 (96.1) | 125 ( 94.7) | 0.415 |  | 98 ( 96.1) | 27 ( 90.0) | 0.207 |
|  | Hand disinfectant use |  | 6629 (82.4) |  | 6519 (82.4) | 110 ( 83.3) | 0.786 |  | 84 ( 82.4) | 26 ( 86.7) | 0.579 |
|  | Cover face while sneezing/coughing |  | 7448 (92.6) |  | 7329 (92.7) | 119 ( 90.2) | 0.275 |  | 93 ( 91.2) | 26 ( 86.7) | 0.469 |
|  | Wearing a mask |  | 7659 (95.2) |  | 7535 (95.3) | 124 ( 93.9) | 0.477 |  | 97 ( 95.1) | 27 ( 90.0) | 0.313 |
|  | Avoiding public transport |  | 6116 (76.1) |  | 6030 (76.2) | 86 ( 65.2) | 0.003 |  | 69 ( 67.6) | 17 ( 56.7) | 0.269 |
|  | Social distancing |  | 7397 (92.0) |  | 7277 (92.0) | 120 ( 90.9) | 0.645 |  | 92 ( 90.2) | 28 ( 93.3) | 0.602 |
|  | Work from home |  | 2927 (36.4) |  | 2883 (36.5) | 44 ( 33.3) | 0.460 |  | 36 ( 35.3) | 8 ( 26.7) | 0.380 |
|  | Avoid travel in general |  | 5577 (69.4) |  | 5492 (69.4) | 85 ( 64.4) | 0.213 |  | 68 ( 66.7) | 17 ( 56.7) | 0.316 |
|  | Self-Isolation |  | 2124 (26.4) |  | 2101 (26.6) | 23 ( 17.4) | 0.020 |  | 19 ( 18.6) | 4 ( 13.3) | 0.504 |
|  | None of these |  | 30 ( 0.4) |  | 27 ( 0.3) | 3 ( 2.3) | 0.002 |  | 2 ( 2.0) | 1 ( 3.3) | 0.661 |
| What COVID-19 symptoms did you have? | |  |  |  |  |  |  |  |  |  |  |
|  | No symptoms |  | 394 (51.8) |  | 387 (61.5) | 7 ( 5.3) | 3.E-17 |  | 7 ( 6.9) | 0 ( 0.0) | 0.991 |
|  | Fever (100.4F or more) |  | 142 (18.7) |  | 63 (10.0) | 79 ( 59.8) | 1.E-31 |  | 54 ( 52.9) | 25 ( 83.3) | 0.005 |
|  | Fatigue |  | 272 (35.7) |  | 168 (26.7) | 104 ( 78.8) | 1.E-23 |  | 80 ( 78.4) | 24 ( 80.0) | 0.853 |
|  | Cough |  | 222 (29.2) |  | 142 (22.6) | 80 ( 60.6) | 2.E-16 |  | 62 ( 60.8) | 18 ( 60.0) | 0.938 |
|  | Runny Nose |  | 121 (15.9) |  | 88 (14.0) | 33 ( 25.0) | 0.002 |  | 31 ( 30.4) | 2 ( 6.7) | 0.018 |
|  | Sneezing |  | 81 (10.6) |  | 59 ( 9.4) | 22 ( 16.7) | 0.015 |  | 19 ( 18.6) | 3 ( 10.0) | 0.273 |
|  | Congestion |  | 128 (16.8) |  | 85 (13.5) | 43 ( 32.6) | 3.E-07 |  | 36 ( 35.3) | 7 ( 23.3) | 0.223 |
|  | Loss of Smell/Taste |  | 89 (11.7) |  | 37 ( 5.9) | 52 ( 39.4) | 2.E-21 |  | 41 ( 40.2) | 11 ( 36.7) | 0.728 |
|  | Shortness of Breath |  | 175 (23.0) |  | 89 (14.1) | 86 ( 65.2) | 2.E-29 |  | 63 ( 61.8) | 23 ( 76.7) | 0.137 |
|  | Chest Pain |  | 100 (13.1) |  | 51 ( 8.1) | 49 ( 37.1) | 3.E-16 |  | 42 ( 41.2) | 7 ( 23.3) | 0.080 |
|  | Muscle Aches |  | 190 (25.0) |  | 102 (16.2) | 88 ( 66.7) | 1.E-27 |  | 70 ( 68.6) | 18 ( 60.0) | 0.380 |
|  | Nausea/Diarrhea |  | 133 (17.5) |  | 73 (11.6) | 60 ( 45.5) | 7.E-18 |  | 47 ( 46.1) | 13 ( 43.3) | 0.791 |
|  | Headache |  | 215 (28.3) |  | 132 (21.0) | 83 ( 62.9) | 2.E-19 |  | 68 ( 66.7) | 15 ( 50.0) | 0.100 |
|  | Sore Throat |  | 173 (22.7) |  | 122 (19.4) | 51 ( 38.6) | 3.E-06 |  | 44 ( 43.1) | 7 ( 23.3) | 0.055 |
| Which of the following things did you do in the two weeks prior to showing symptoms? | |  |  |  |  |  |  |  |  |  |  |
|  | None of these things |  | 49 (13.4) |  | 33 (13.6) | 16 ( 12.8) | 0.823 |  | 9 ( 9.5) | 7 ( 23.3) | 0.055 |
|  | Participate in any Festivals/Events of over 50 people |  | 40 (10.9) |  | 29 (12.0) | 11 ( 8.8) | 0.356 |  | 7 ( 7.4) | 4 ( 13.3) | 0.321 |
|  | Go to the Grocery Store |  | 262 (71.4) |  | 173 (71.5) | 89 ( 71.2) | 0.954 |  | 73 ( 76.8) | 16 ( 53.3) | 0.015 |
|  | Work in direct contact with the public (e.g. grocery store employee, cashier, clerk, etc.) |  | 135 (36.8) |  | 87 (36.0) | 48 ( 38.4) | 0.645 |  | 36 ( 37.9) | 12 ( 40.0) | 0.836 |
|  | Travel within your State |  | 58 (15.8) |  | 46 (19.0) | 12 ( 9.6) | 0.022 |  | 11 ( 11.6) | 1 ( 3.3) | 0.211 |
|  | Travel Domestically (Between States) |  | 62 (16.9) |  | 44 (18.2) | 18 ( 14.4) | 0.360 |  | 15 ( 15.8) | 3 ( 10.0) | 0.435 |
|  | Go to a Bar/Restaurant |  | 144 (39.2) |  | 101 (41.7) | 43 ( 34.4) | 0.173 |  | 34 ( 35.8) | 9 ( 30.0) | 0.561 |
|  | Travel Internationally |  | 15 ( 4.1) |  | 13 ( 5.4) | 2 ( 1.6) | 0.103 |  | 2 ( 2.1) | 0 ( 0.0) | 0.989 |
| Are you currently working as an essential employee? (NOT from home) If so, please select from the options below: | |  |  |  |  |  |  |  |  |  |  |
|  | No - I am not currently working as an essential employee |  | 6433 (80.2) |  | 6360 (80.6) | 73 ( 55.3) | 9.E-12 |  | 56 ( 54.9) | 17 ( 56.7) | 0.864 |
|  | Grocery/convenience store employee |  | 58 ( 0.7) |  | 55 ( 0.7) | 3 ( 2.3) | 0.046 |  | 2 ( 2.0) | 1 ( 3.3) | 0.661 |
|  | Warehouse/factory worker |  | 96 ( 1.2) |  | 94 ( 1.2) | 2 ( 1.5) | 0.735 |  | 2 ( 2.0) | 0 ( 0.0) | 0.989 |
|  | First responder (EMT, Firefighter, police, military) |  | 49 ( 0.6) |  | 46 ( 0.6) | 3 ( 2.3) | 0.022 |  | 3 ( 2.9) | 0 ( 0.0) | 0.991 |
|  | Medical professional/staff member (doctor, nurse, clerk, janitorial staff, etc.) |  | 665 ( 8.3) |  | 633 ( 8.0) | 32 ( 24.2) | 4.E-10 |  | 24 ( 23.5) | 8 ( 26.7) | 0.725 |
|  | Truck driver/delivery |  | 28 ( 0.3) |  | 28 ( 0.4) | 0 ( 0.0) | 0.978 |  | 0 ( 0.0) | 0 ( 0.0) |  |
|  | Bus driver/train operator |  | 6 ( 0.1) |  | 6 ( 0.1) | 0 ( 0.0) | 0.977 |  | 0 ( 0.0) | 0 ( 0.0) |  |
|  | Restaurant worker |  | 51 ( 0.6) |  | 49 ( 0.6) | 2 ( 1.5) | 0.215 |  | 2 ( 2.0) | 0 ( 0.0) | 0.989 |
|  | Sanitation worker |  | 12 ( 0.1) |  | 11 ( 0.1) | 1 ( 0.8) | 0.105 |  | 0 ( 0.0) | 1 ( 3.3) | 0.991 |
| Please select all the immune system conditions that apply to you.* | |  |  |  |  |  |  |  |  |  |  |
|  | I have none of these conditions |  | 5555 (69.1) |  | 5468 (69.1) | 87 ( 65.9) | 0.242 |  | 68 ( 66.7) | 19 ( 63.3) | 0.974 |
|  | Type II Diabetes (high blood sugar) |  | 950 (11.8) |  | 930 (11.8) | 20 ( 15.2) | 0.020 |  | 13 ( 12.7) | 7 ( 23.3) | 0.610 |
|  | Immunocompromised status |  | 687 ( 8.5) |  | 675 ( 8.5) | 12 ( 9.1) | 0.865 |  | 9 ( 8.8) | 3 ( 10.0) | 0.929 |
|  | Autoimmune or rheumatologic disease |  | 984 (12.2) |  | 961 (12.2) | 23 ( 17.4) | 0.075 |  | 21 ( 20.6) | 2 ( 6.7) | 0.166 |
|  | Organ transplant |  | 164 ( 2.0) |  | 161 ( 2.0) | 3 ( 2.3) | 0.920 |  | 1 ( 1.0) | 2 ( 6.7) | 0.130 |
|  | Type I Diabetes (high blood sugar) |  | 249 ( 3.1) |  | 248 ( 3.1) | 1 ( 0.8) | 0.085 |  | 1 ( 1.0) | 0 ( 0.0) | 0.992 |
|  | HIV |  | 30 ( 0.4) |  | 29 ( 0.4) | 1 ( 0.8) | 0.510 |  | 0 ( 0.0) | 1 ( 3.3) | 0.991 |
|  | Bone marrow transplant |  | 19 ( 0.2) |  | 19 ( 0.2) | 0 ( 0.0) | 0.973 |  | 0 ( 0.0) | 0 ( 0.0) |  |
| Please select all respiratory conditions that apply to you.* | |  |  |  |  |  |  |  |  |  |  |
|  | I have none of these conditions |  | 5138 (63.9) |  | 5062 (64.0) | 76 ( 57.6) | 0.033 |  | 60 ( 58.8) | 16 ( 53.3) | 0.675 |
|  | Sleep Apnea |  | 1779 (22.1) |  | 1750 (22.1) | 29 ( 22.0) | 0.395 |  | 20 ( 19.6) | 9 ( 30.0) | 0.714 |
|  | I use a home CPAP |  | 1408 (17.5) |  | 1385 (17.5) | 23 ( 17.4) | 0.391 |  | 15 ( 14.7) | 8 ( 26.7) | 0.444 |
|  | Asthma |  | 1172 (14.6) |  | 1146 (14.5) | 26 ( 19.7) | 0.224 |  | 24 ( 23.5) | 2 ( 6.7) | 0.066 |
|  | Chronic Obstructive Pulmonary Disease (COPD) |  | 309 ( 3.8) |  | 302 ( 3.8) | 7 ( 5.3) | 0.065 |  | 3 ( 2.9) | 4 ( 13.3) | 0.359 |
|  | Emphysema |  | 94 ( 1.2) |  | 90 ( 1.1) | 4 ( 3.0) | 0.006 |  | 0 ( 0.0) | 4 ( 13.3) | 0.988 |
|  | Cystic Fibrosis |  | 6 ( 0.1) |  | 5 ( 0.1) | 1 ( 0.8) | 0.046 |  | 1 ( 1.0) | 0 ( 0.0) | 0.992 |
| Please select all the genitourinary/metabolic conditions that apply to you.* | |  |  |  |  |  |  |  |  |  |  |
|  | I have none of these conditions |  | 7202 (89.6) |  | 7084 (89.6) | 118 ( 89.4) | 0.511 |  | 90 ( 88.2) | 28 ( 93.3) | 0.194 |
|  | Chronic Kidney Disease |  | 518 ( 6.4) |  | 511 ( 6.5) | 7 ( 5.3) | 0.981 |  | 5 ( 4.9) | 2 ( 6.7) | 0.989 |
|  | Liver Disease |  | 199 ( 2.5) |  | 193 ( 2.4) | 6 ( 4.5) | 0.097 |  | 6 ( 5.9) | 0 ( 0.0) | 0.991 |
|  | Gallbladder Disease |  | 132 ( 1.6) |  | 130 ( 1.6) | 2 ( 1.5) | 0.992 |  | 2 ( 2.0) | 0 ( 0.0) | 0.988 |
|  | Pancreas Disease |  | 75 ( 0.9) |  | 75 ( 0.9) | 0 ( 0.0) | 0.976 |  | 0 ( 0.0) | 0 ( 0.0) |  |
| Please select all the cardiovascular conditions that apply to you.* | |  |  |  |  |  |  |  |  |  |  |
|  | I have none of these conditions |  | 4585 (57.0) |  | 4502 (56.9) | 83 ( 62.9) | 0.434 |  | 64 ( 62.7) | 19 ( 63.3) | 0.143 |
|  | Stroke |  | 219 ( 2.7) |  | 215 ( 2.7) | 4 ( 3.0) | 0.404 |  | 4 ( 3.9) | 0 ( 0.0) | 0.988 |
|  | Hypertension (high blood pressure) |  | 2622 (32.6) |  | 2587 (32.7) | 35 ( 26.5) | 0.682 |  | 26 ( 25.5) | 9 ( 30.0) | 0.498 |
|  | Balloon angioplasty or percutaneuous coronary intervention |  | 299 ( 3.7) |  | 298 ( 3.8) | 1 ( 0.8) | 0.209 |  | 1 ( 1.0) | 0 ( 0.0) | 0.991 |
|  | Arrythmias |  | 643 ( 8.0) |  | 631 ( 8.0) | 12 ( 9.1) | 0.170 |  | 10 ( 9.8) | 2 ( 6.7) | 0.329 |
|  | Coronary artery bypass |  | 228 ( 2.8) |  | 227 ( 2.9) | 1 ( 0.8) | 0.391 |  | 0 ( 0.0) | 1 ( 3.3) | 0.991 |
|  | Myocardial infarction |  | 210 ( 2.6) |  | 208 ( 2.6) | 2 ( 1.5) | 0.712 |  | 1 ( 1.0) | 1 ( 3.3) | 0.452 |
|  | Congestive heart failure |  | 304 ( 3.8) |  | 296 ( 3.7) | 8 ( 6.1) | 0.033 |  | 6 ( 5.9) | 2 ( 6.7) | 0.643 |
|  | Peripheral vascular disease |  | 173 ( 2.2) |  | 171 ( 2.2) | 2 ( 1.5) | 0.937 |  | 0 ( 0.0) | 2 ( 6.7) | 0.987 |
|  | Blood clot or clotting disorder |  | 331 ( 4.1) |  | 325 ( 4.1) | 6 ( 4.5) | 0.631 |  | 6 ( 5.9) | 0 ( 0.0) | 0.991 |
| Please select all the neurological conditions that apply to you.* | |  |  |  |  |  |  |  |  |  |  |
|  | I have neither of these conditions |  | 7771 (96.6) |  | 7645 (96.7) | 126 ( 95.5) | 0.304 |  | 100 ( 98.0) | 26 ( 86.7) | 0.161 |
|  | Neurological disease |  | 255 ( 3.2) |  | 251 ( 3.2) | 4 ( 3.0) | 0.934 |  | 2 ( 2.0) | 2 ( 6.7) | 0.834 |
|  | Dementia |  | 27 ( 0.3) |  | 25 ( 0.3) | 2 ( 1.5) | 0.008 |  | 0 ( 0.0) | 2 ( 6.7) | 0.987 |
| Please select all the conditions/treatments that apply to you.* | |  |  |  |  |  |  |  |  |  |  |
|  | I have none of these conditions |  | 6976 (86.8) |  | 6854 (86.7) | 122 ( 92.4) | 0.241 |  | 95 ( 93.1) | 27 ( 90.0) | 0.895 |
|  | Malignant solid tumor |  | 378 ( 4.7) |  | 374 ( 4.7) | 4 ( 3.0) | 0.635 |  | 4 ( 3.9) | 0 ( 0.0) | 0.989 |
|  | Chemotherapy |  | 516 ( 6.4) |  | 511 ( 6.5) | 5 ( 3.8) | 0.427 |  | 3 ( 2.9) | 2 ( 6.7) | 0.861 |
|  | Radiation Therapy |  | 641 ( 8.0) |  | 636 ( 8.0) | 5 ( 3.8) | 0.222 |  | 3 ( 2.9) | 2 ( 6.7) | 0.270 |
|  | Lymphoma |  | 99 ( 1.2) |  | 99 ( 1.3) | 0 ( 0.0) | 0.973 |  | 0 ( 0.0) | 0 ( 0.0) |  |
|  | Leukemia |  | 51 ( 0.6) |  | 51 ( 0.6) | 0 ( 0.0) | 0.971 |  | 0 ( 0.0) | 0 ( 0.0) |  |
| *Models to evaluate self-reported health conditions were adjusted for age and sex | | | | | | | | | | | |
