# Supplementary material for: Exposure and risk factors for COVID-19 and the impact of staying home on Michigan residents
Source: PLoS One. 2021 Feb 8;16(2):e0246447. doi: 10.1371/journal.pone.0246447 (PMC7870003; doi:10.1371/journal.pone.0246447)
Supplement: S7 Table — (DOCX) [file pone.0246447.s007.docx]

| **Table S7.** Differences in clinical and social risk factors between African American and European American survey respondents | | | | | | | |
| --- | --- | --- | --- | --- | --- | --- | --- |
|  |  |  | **Overall** |  | **Race** | |  |
|  |  |  |  |  | **AA** | **EA** | **p** |
|  |  | n | 8041 |  | 233 | 7387 |  |
| Sex | |  |  |  |  |  |  |
|  | F |  | 4661 (58.5) |  | 161 ( 69.4) | 4278 ( 58.3) | 0.001 |
|  | M |  | 3310 (41.5) |  | 71 ( 30.6) | 3061 ( 41.7) |  |
| Age | |  |  |  |  |  |  |
|  |  |  | 59.15 (14.79) |  | 53.29 (14.90) | 59.52 (14.71) | 1.E-09 |
| BMI | |  |  |  |  |  |  |
|  |  |  | 29.12 (6.64) |  | 32.52 (8.11) | 29.02 (6.55) | 9.E-10 |
| BMI category | |  |  |  |  |  |  |
|  | underweight |  | 73 ( 0.9) |  | 1 ( 0.4) | 68 ( 1.0) | 4.E-09 |
|  | normal |  | 2179 (28.2) |  | 35 ( 15.6) | 2030 ( 28.5) |  |
|  | overweight |  | 2582 (33.5) |  | 59 ( 26.3) | 2396 ( 33.7) |  |
|  | obese |  | 2881 (37.3) |  | 129 ( 57.6) | 2622 ( 36.8) |  |
| Education | |  |  |  |  |  |  |
|  | High school or less |  | 1365 (17.1) |  | 39 ( 16.8) | 1262 ( 17.2) | 0.604 |
|  | Associate degree |  | 1279 (16.0) |  | 43 ( 18.5) | 1173 ( 15.9) |  |
|  | Bachelor's degree |  | 2492 (31.2) |  | 65 ( 28.0) | 2309 ( 31.4) |  |
|  | Advanced degree |  | 2860 (35.8) |  | 85 ( 36.6) | 2613 ( 35.5) |  |
| Income | |  |  |  |  |  |  |
|  | <40 |  | 1070 (14.1) |  | 64 ( 28.2) | 933 ( 13.4) | 1.E-09 |
|  | 40-100 |  | 3300 (43.6) |  | 89 ( 39.2) | 3068 ( 44.0) |  |
|  | >100 |  | 3201 (42.3) |  | 74 ( 32.6) | 2972 ( 42.6) |  |
| Were you admitted to the hospital due to COVID-19 symptoms? | |  |  |  |  |  |  |
|  | Yes |  | 32 ( 4.2) |  | 7 ( 18.9) | 21 ( 3.1) | 1.E-05 |
|  | No |  | 729 (95.8) |  | 30 ( 81.1) | 660 ( 96.9) |  |
| What is your current living situation? - Selected Choice | |  |  |  |  |  |  |
|  | A family member owns the house I live in |  | 465 ( 5.8) |  | 20 ( 8.6) | 416 ( 5.7) | 3.E-29 |
|  | I own a house |  | 6546 (81.9) |  | 136 ( 58.4) | 6096 ( 82.9) |  |
|  | I rent a house/apartment |  | 794 ( 9.9) |  | 73 ( 31.3) | 665 ( 9.0) |  |
|  | Other |  | 191 ( 2.4) |  | 4 ( 1.7) | 180 ( 2.4) |  |
| Have any of your family members been diagnosed with COVID-19 within two weeks after you saw them last? | |  |  |  |  |  |  |
|  | Yes |  | 44 (15.5) |  | 9 ( 39.1) | 33 ( 13.5) | 0.005 |
|  | No |  | 219 (77.1) |  | 12 ( 52.2) | 193 ( 79.1) |  |
|  | Unsure |  | 21 ( 7.4) |  | 2 ( 8.7) | 18 ( 7.4) |  |
| Has anyone outside of your household been diagnosed with COVID-19 within two weeks after you saw them last? | |  |  |  |  |  |  |
|  | Yes |  | 38 (13.5) |  | 7 ( 38.9) | 27 ( 11.0) | 0.003 |
|  | No |  | 150 (53.4) |  | 6 ( 33.3) | 134 ( 54.5) |  |
|  | Unsure |  | 93 (33.1) |  | 5 ( 27.8) | 85 ( 34.6) |  |
| Approximately how many days did your symptoms last? (time from symptom onset to completely recovered) | |  |  |  |  |  |  |
|  |  |  | 14.22 (10.90) |  | 15.35 (10.24) | 14.13 (10.98) | 0.566 |
| Please indicate your physical activity participation in relation to shelter at home recommendations. | |  |  |  |  |  |  |
|  | Before |  | 3.68 (1.85) |  | 3.27 (1.83) | 3.69 (1.85) | 0.003 |
|  | After |  | 3.74 (2.05) |  | 3.14 (1.88) | 3.75 (2.05) | 6.E-05 |
| What precautions are you taking to protect yourself or others from COVID-19? | |  |  |  |  |  |  |
|  | Frequent hand washing |  | 7725 (96.1) |  | 227 ( 97.4) | 7093 ( 96.0) | 0.360 |
|  | Hand disinfectant use |  | 6629 (82.4) |  | 208 ( 89.3) | 6070 ( 82.2) | 0.007 |
|  | Cover face while sneezing/coughing |  | 7448 (92.6) |  | 214 ( 91.8) | 6855 ( 92.8) | 0.671 |
|  | Wearing a mask |  | 7659 (95.2) |  | 228 ( 97.9) | 7039 ( 95.3) | 0.094 |
|  | Avoiding public transport |  | 6116 (76.1) |  | 179 ( 76.8) | 5615 ( 76.0) | 0.835 |
|  | Social distancing (but not from other members of the household) |  | 7397 (92.0) |  | 215 ( 92.3) | 6799 ( 92.0) | 0.994 |
|  | Work from home |  | 2927 (36.4) |  | 80 ( 34.3) | 2664 ( 36.1) | 0.637 |
|  | Avoid travel in general |  | 5577 (69.4) |  | 158 ( 67.8) | 5120 ( 69.3) | 0.677 |
|  | Self-Isolation |  | 2124 (26.4) |  | 74 ( 31.8) | 1939 ( 26.2) | 0.071 |
|  | None of these |  | 30 ( 0.4) |  | 0 ( 0.0) | 28 ( 0.4) | 0.695 |
| How were you exposed to COVID-19/suspected COVID-19? | |  |  |  |  |  |  |
|  | Coworker was sick |  | 133 (21.2) |  | 5 ( 20.8) | 126 ( 22.1) | 1.000 |
|  | Work in high risk environment (first responder, hospital employee) |  | 205 (32.7) |  | 8 ( 33.3) | 187 ( 32.7) | 1.000 |
|  | Family member was sick |  | 193 (30.8) |  | 13 ( 54.2) | 169 ( 29.6) | 0.020 |
|  | Friend or other social contact was sick |  | 94 (15.0) |  | 3 ( 12.5) | 86 ( 15.1) | 0.958 |
|  | Travel related |  | 42 ( 6.7) |  | 1 ( 4.2) | 40 ( 7.0) | 0.899 |
|  | Work in a service job (grocery store employee, sanitation worker, warehouse workers, etc.) |  | 38 ( 6.1) |  | 0 ( 0.0) | 34 ( 6.0) | 0.434 |
|  | Unsure |  | 17 ( 2.7) |  | 0 ( 0.0) | 15 ( 2.6) | 0.889 |
| What COVID-19 symptoms did you have? | |  |  |  |  |  |  |
|  | No symptoms |  | 394 (51.8) |  | 11 ( 29.7) | 362 ( 53.2) | 0.009 |
|  | Fever (100.4F or more) |  | 142 (18.7) |  | 12 ( 32.4) | 120 ( 17.6) | 0.041 |
|  | Fatigue |  | 272 (35.7) |  | 21 ( 56.8) | 237 ( 34.8) | 0.011 |
|  | Cough |  | 222 (29.2) |  | 14 ( 37.8) | 192 ( 28.2) | 0.282 |
|  | Runny Nose |  | 121 (15.9) |  | 7 ( 18.9) | 106 ( 15.6) | 0.754 |
|  | Sneezing |  | 81 (10.6) |  | 5 ( 13.5) | 71 ( 10.4) | 0.749 |
|  | Congestion |  | 128 (16.8) |  | 13 ( 35.1) | 106 ( 15.6) | 0.004 |
|  | Loss of Smell/Taste |  | 89 (11.7) |  | 7 ( 18.9) | 75 ( 11.0) | 0.227 |
|  | Shortness of Breath |  | 175 (23.0) |  | 11 ( 29.7) | 151 ( 22.2) | 0.385 |
|  | Chest Pain |  | 100 (13.1) |  | 7 ( 18.9) | 88 ( 12.9) | 0.424 |
|  | Muscle Aches |  | 190 (25.0) |  | 17 ( 45.9) | 162 ( 23.8) | 0.005 |
|  | Nausea/Diarrhea |  | 133 (17.5) |  | 8 ( 21.6) | 115 ( 16.9) | 0.603 |
|  | Headache |  | 215 (28.3) |  | 16 ( 43.2) | 184 ( 27.0) | 0.051 |
|  | Sore Throat |  | 173 (22.7) |  | 14 ( 37.8) | 147 ( 21.6) | 0.035 |
| Which of the following things did you do in the two weeks prior to showing symptoms? | |  |  |  |  |  |  |
|  | None of these things |  | 49 (13.4) |  | 4 ( 15.4) | 41 ( 12.9) | 0.948 |
|  | Participate in any Festivals/Events of over 50 people |  | 40 (10.9) |  | 2 ( 7.7) | 37 ( 11.6) | 0.777 |
|  | Go to the Grocery Store |  | 262 (71.4) |  | 19 ( 73.1) | 230 ( 72.1) | 1.000 |
|  | Work in direct contact with the public (e.g. grocery store employee, cashier, clerk, etc.) |  | 135 (36.8) |  | 8 ( 30.8) | 117 ( 36.7) | 0.696 |
|  | Travel within your State |  | 58 (15.8) |  | 4 ( 15.4) | 50 ( 15.7) | 1.000 |
|  | Travel Domestically (Between States) |  | 62 (16.9) |  | 0 ( 0.0) | 59 ( 18.5) | 0.033 |
|  | Go to a Bar/Restaurant |  | 144 (39.2) |  | 6 ( 23.1) | 133 ( 41.7) | 0.098 |
|  | Travel Internationally |  | 15 ( 4.1) |  | 0 ( 0.0) | 13 ( 4.1) | 0.607 |
| Are you currently working as an essential employee? (NOT from home) If so, please select from the options below: | |  |  |  |  |  |  |
|  | No - I am not currently working as an essential employee |  | 6433 (80.2) |  | 170 ( 73.3) | 5945 ( 80.6) | 0.007 |
|  | Grocery/convenience store employee |  | 58 ( 0.7) |  | 3 ( 1.3) | 50 ( 0.7) | 0.479 |
|  | Warehouse/factory worker |  | 96 ( 1.2) |  | 4 ( 1.7) | 84 ( 1.1) | 0.611 |
|  | First responder (EMT, Firefighter, police, military) |  | 49 ( 0.6) |  | 2 ( 0.9) | 43 ( 0.6) | 0.912 |
|  | Medical professional/staff member (doctor, nurse, clerk, janitorial staff, etc.) |  | 665 ( 8.3) |  | 32 ( 13.8) | 589 ( 8.0) | 0.002 |
|  | Truck driver/delivery |  | 28 ( 0.3) |  | 0 ( 0.0) | 27 ( 0.4) | 0.717 |
|  | Bus driver/train operator |  | 6 ( 0.1) |  | 1 ( 0.4) | 5 ( 0.1) | 0.452 |
|  | Restaurant worker |  | 51 ( 0.6) |  | 1 ( 0.4) | 47 ( 0.6) | 1.000 |
|  | Sanitation worker |  | 12 ( 0.1) |  | 0 ( 0.0) | 10 ( 0.1) | 1.000 |
| Please select all the immune system conditions that apply to you. | |  |  |  |  |  |  |
|  | I have none of these conditions |  | 5555 (69.1) |  | 131 ( 56.2) | 5144 ( 69.6) | 2.E-05 |
|  | Type II Diabetes (high blood sugar) |  | 950 (11.8) |  | 62 ( 26.6) | 827 ( 11.2) | 1.E-12 |
|  | Immunocompromised status |  | 687 ( 8.5) |  | 18 ( 7.7) | 637 ( 8.6) | 0.717 |
|  | Autoimmune or rheumatologic disease |  | 984 (12.2) |  | 38 ( 16.3) | 898 ( 12.2) | 0.072 |
|  | Organ transplant |  | 164 ( 2.0) |  | 8 ( 3.4) | 145 ( 2.0) | 0.181 |
|  | Type I Diabetes (high blood sugar) |  | 249 ( 3.1) |  | 7 ( 3.0) | 231 ( 3.1) | 1.000 |
|  | HIV |  | 30 ( 0.4) |  | 3 ( 1.3) | 27 ( 0.4) | 0.093 |
|  | Bone marrow transplant |  | 19 ( 0.2) |  | 2 ( 0.9) | 16 ( 0.2) | 0.193 |
| Please select all respiratory conditions that apply to you. | |  |  |  |  |  |  |
|  | I have none of these conditions |  | 5138 (63.9) |  | 127 ( 54.5) | 4740 ( 64.2) | 0.003 |
|  | Sleep Apnea |  | 1779 (22.1) |  | 70 ( 30.0) | 1619 ( 21.9) | 0.004 |
|  | I use a home CPAP |  | 1408 (17.5) |  | 54 ( 23.2) | 1280 ( 17.3) | 0.026 |
|  | Asthma |  | 1172 (14.6) |  | 49 ( 21.0) | 1065 ( 14.4) | 0.007 |
|  | Chronic Obstructive Pulmonary Disease (COPD) |  | 309 ( 3.8) |  | 7 ( 3.0) | 279 ( 3.8) | 0.663 |
|  | Emphysema |  | 94 ( 1.2) |  | 0 ( 0.0) | 85 ( 1.2) | 0.184 |
|  | Cystic Fibrosis |  | 6 ( 0.1) |  | 0 ( 0.0) | 5 ( 0.1) | 1.000 |
| Please select all the genitourinary/metabolic conditions that apply to you. | |  |  |  |  |  |  |
|  | I have none of these conditions |  | 7202 (89.6) |  | 196 ( 84.1) | 6646 ( 90.0) | 0.005 |
|  | Chronic Kidney Disease |  | 518 ( 6.4) |  | 32 ( 13.7) | 447 ( 6.1) | 4.E-06 |
|  | Liver Disease |  | 199 ( 2.5) |  | 6 ( 2.6) | 176 ( 2.4) | 1.000 |
|  | Gallbladder Disease |  | 132 ( 1.6) |  | 3 ( 1.3) | 118 ( 1.6) | 0.915 |
|  | Pancreas Disease |  | 75 ( 0.9) |  | 3 ( 1.3) | 70 ( 0.9) | 0.855 |
| Please select all the cardiovascular conditions that apply to you. | |  |  |  |  |  |  |
|  | I have none of these conditions |  | 4585 (57.0) |  | 118 ( 50.6) | 4212 ( 57.0) | 0.062 |
|  | Stroke |  | 219 ( 2.7) |  | 7 ( 3.0) | 195 ( 2.6) | 0.893 |
|  | Hypertension (high blood pressure) |  | 2622 (32.6) |  | 105 ( 45.1) | 2390 ( 32.4) | 6.E-05 |
|  | Balloon angioplasty or percutaneuous coronary intervention |  | 299 ( 3.7) |  | 5 ( 2.1) | 277 ( 3.7) | 0.271 |
|  | Arrythmias |  | 643 ( 8.0) |  | 12 ( 5.2) | 596 ( 8.1) | 0.135 |
|  | Coronary artery bypass |  | 228 ( 2.8) |  | 6 ( 2.6) | 212 ( 2.9) | 0.947 |
|  | Myocardial infarction |  | 210 ( 2.6) |  | 2 ( 0.9) | 196 ( 2.7) | 0.137 |
|  | Congestive heart failure |  | 304 ( 3.8) |  | 10 ( 4.3) | 277 ( 3.7) | 0.800 |
|  | Peripheral vascular disease |  | 173 ( 2.2) |  | 4 ( 1.7) | 156 ( 2.1) | 0.856 |
|  | Blood clot or clotting disorder |  | 331 ( 4.1) |  | 12 ( 5.2) | 305 ( 4.1) | 0.547 |
| Please select all the neurological conditions that apply to you. | |  |  |  |  |  |  |
|  | I have neither of these conditions |  | 7771 (96.6) |  | 228 ( 97.9) | 7142 ( 96.7) | 0.423 |
|  | Neurological disease |  | 255 ( 3.2) |  | 6 ( 2.6) | 231 ( 3.1) | 0.775 |
|  | Dementia |  | 27 ( 0.3) |  | 0 ( 0.0) | 24 ( 0.3) | 0.781 |
| Please select all the conditions/treatments that apply to you. | |  |  |  |  |  |  |
|  | I have none of these conditions |  | 6976 (86.8) |  | 211 ( 90.6) | 6397 ( 86.6) | 0.098 |
|  | Malignant solid tumor |  | 378 ( 4.7) |  | 2 ( 0.9) | 351 ( 4.8) | 0.009 |
|  | Chemotherapy |  | 516 ( 6.4) |  | 12 ( 5.2) | 477 ( 6.5) | 0.506 |
|  | Radiation Therapy |  | 641 ( 8.0) |  | 14 ( 6.0) | 596 ( 8.1) | 0.309 |
|  | Lymphoma |  | 99 ( 1.2) |  | 2 ( 0.9) | 89 ( 1.2) | 0.863 |
|  | Leukemia |  | 51 ( 0.6) |  | 0 ( 0.0) | 47 ( 0.6) | 0.426 |
