# Supplementary material for: Exposure and risk factors for COVID-19 and the impact of staying home on Michigan residents
Source: PLoS One. 2021 Feb 8;16(2):e0246447. doi: 10.1371/journal.pone.0246447 (PMC7870003; doi:10.1371/journal.pone.0246447)
Supplement: S8 Table — (DOCX) [file pone.0246447.s008.docx]

| **Table S8.** Behavioral change by sex | | | | | | | |
| --- | --- | --- | --- | --- | --- | --- | --- |
|  |  |  | **Overall** |  | **Sex** | |  |
|  |  |  |  |  | **Female** | **Male** | **p** |
|  |  | n | 7909 |  | 4582 | 3258 |  |
| I have increased moderate to strenuous exercise | |  |  |  |  |  |  |
|  | Disagree |  | 2921 (38.6) |  | 1801 (41.2) | 1090 (34.8) | 2.E-04 |
|  | Same |  | 2907 (38.4) |  | 1564 (35.7) | 1320 (42.2) |  |
|  | Agree |  | 1744 (23.0) |  | 1011 (23.1) | 719 (23.0) |  |
| I have increased my alcohol consumption | |  |  |  |  |  |  |
|  | Disagree |  | 3530 (52.7) |  | 2059 (53.3) | 1438 (51.8) | 0.498 |
|  | Same |  | 1949 (29.1) |  | 1029 (26.7) | 911 (32.8) |  |
|  | Agree |  | 1219 (18.2) |  | 773 (20.0) | 429 (15.4) |  |
| I have increased my drug use | |  |  |  |  |  |  |
|  | Disagree |  | 854 (68.3) |  | 518 (71.6) | 324 (64.0) | 0.012 |
|  | Same |  | 237 (19.0) |  | 118 (16.3) | 117 (23.1) |  |
|  | Agree |  | 159 (12.7) |  | 87 (12.0) | 65 (12.8) |  |
| I have increased my tobacco use | |  |  |  |  |  |  |
|  | Disagree |  | 121 (28.2) |  | 61 (24.9) | 60 (33.0) | 0.001 |
|  | Same |  | 154 (35.9) |  | 77 (31.4) | 76 (41.8) |  |
|  | Agree |  | 154 (35.9) |  | 107 (43.7) | 46 (25.3) |  |
| I have improved my sleep habits | |  |  |  |  |  |  |
|  | Disagree |  | 2297 (30.1) |  | 1470 (33.2) | 802 (25.6) | 1.E-05 |
|  | Same |  | 4170 (54.7) |  | 2263 (51.1) | 1878 (60.1) |  |
|  | Agree |  | 1159 (15.2) |  | 699 (15.8) | 447 (14.3) |  |
| I have improved my nutrition (Dietary Habits) | |  |  |  |  |  |  |
|  | Disagree |  | 1780 (23.1) |  | 1183 (26.4) | 576 (18.3) | 4.E-12 |
|  | Same |  | 3914 (50.8) |  | 2188 (48.8) | 1692 (53.7) |  |
|  | Agree |  | 2007 (26.1) |  | 1109 (24.8) | 885 (28.1) |  |
| I have gained weight | |  |  |  |  |  |  |
|  | Disagree |  | 2486 (32.4) |  | 1330 (29.9) | 1135 (35.9) | 6.E-21 |
|  | Same |  | 2743 (35.7) |  | 1493 (33.5) | 1231 (39.0) |  |
|  | Agree |  | 2454 (31.9) |  | 1632 (36.6) | 794 (25.1) |  |
| How concerned have you been about the novel COVID-19 pandemic in the past 7 days? | |  |  |  |  |  |  |
|  |  |  | 5.57 (2.95) |  | 5.69 (2.88) | 5.39 (3.05) | 7.E-06 |
| How concerned are you about - Contracting COVID-19 | |  |  |  |  |  |  |
|  | Not-to-slightly concerned |  | 4162 (52.6) |  | 2408 (52.6) | 1710 (52.5) | 0.953 |
|  | Very-to-extremely concerned |  | 3747 (47.4) |  | 2174 (47.4) | 1548 (47.5) |  |
| How concerned are you about - Someone close to you contracting COVID-19 | |  |  |  |  |  |  |
|  | Not-to-slightly concerned |  | 2983 (37.7) |  | 1632 (35.6) | 1323 (40.6) | 7.E-06 |
|  | Very-to-extremely concerned |  | 4926 (62.3) |  | 2950 (64.4) | 1935 (59.4) |  |
| How concerned are you about - Getting into serious financial trouble | |  |  |  |  |  |  |
|  | Not-to-slightly concerned |  | 6461 (81.7) |  | 3654 (79.7) | 2758 (84.7) | 3.E-08 |
|  | Very-to-extremely concerned |  | 1448 (18.3) |  | 928 (20.3) | 500 (15.3) |  |
| How concerned are you about - Losing your job | |  |  |  |  |  |  |
|  | Not-to-slightly concerned |  | 7071 (89.4) |  | 4015 (87.6) | 2995 (91.9) | 1.E-09 |
|  | Very-to-extremely concerned |  | 838 (10.6) |  | 567 (12.4) | 263 ( 8.1) |  |
| How concerned are you about - That it will be a long time before your life returns to normal | |  |  |  |  |  |  |
|  | Not-to-slightly concerned |  | 3841 (48.6) |  | 2069 (45.2) | 1739 (53.4) | 8.E-13 |
|  | Very-to-extremely concerned |  | 4068 (51.4) |  | 2513 (54.8) | 1519 (46.6) |  |
| How concerned are you about - Not seeing friends and family | |  |  |  |  |  |  |
|  | Not-to-slightly concerned |  | 3673 (46.4) |  | 1979 (43.2) | 1657 (50.9) | 2.E-11 |
|  | Very-to-extremely concerned |  | 4236 (53.6) |  | 2603 (56.8) | 1601 (49.1) |  |
