# Supplementary material for: Exposure and risk factors for COVID-19 and the impact of staying home on Michigan residents
Source: PLoS One. 2021 Feb 8;16(2):e0246447. doi: 10.1371/journal.pone.0246447 (PMC7870003; doi:10.1371/journal.pone.0246447)
Supplement: S9 Table — (DOCX) [file pone.0246447.s009.docx]

| **Table S9.** Behavioral change by income | | | | | | | | |
| --- | --- | --- | --- | --- | --- | --- | --- | --- |
|  |  |  | **Overall** |  | **Income** | | |  |
|  |  |  |  |  | **<40** | **40-100** | **>100** | **p** |
|  |  | n | 7909 |  | 1055 | 3249 | 3137 |  |
| I have increased moderate to strenuous exercise | |  |  |  |  |  |  |  |
|  | Disagree |  | 2921 (38.6) |  | 443 (46.6) | 1237 (39.7) | 1085 (35.3) | 2.E-17 |
|  | Same |  | 2907 (38.4) |  | 345 (36.3) | 1271 (40.8) | 1116 (36.4) |  |
|  | Agree |  | 1744 (23.0) |  | 163 (17.1) | 604 (19.4) | 869 (28.3) |  |
| I have increased my alcohol consumption | |  |  |  |  |  |  |  |
|  | Disagree |  | 3530 (52.7) |  | 539 (68.1) | 1514 (56.2) | 1258 (44.5) | 5.E-38 |
|  | Same |  | 1949 (29.1) |  | 146 (18.4) | 770 (28.6) | 911 (32.2) |  |
|  | Agree |  | 1219 (18.2) |  | 107 (13.5) | 408 (15.2) | 661 (23.4) |  |
| I have increased my drug use | |  |  |  |  |  |  |  |
|  | Disagree |  | 854 (68.3) |  | 164 (69.2) | 383 (67.3) | 277 (68.1) | 0.871 |
|  | Same |  | 237 (19.0) |  | 40 (16.9) | 108 (19.0) | 83 (20.4) |  |
|  | Agree |  | 159 (12.7) |  | 33 (13.9) | 78 (13.7) | 47 (11.5) |  |
| I have increased my tobacco use | |  |  |  |  |  |  |  |
|  | Disagree |  | 121 (28.2) |  | 23 (20.5) | 57 (29.4) | 33 (31.1) | 0.077 |
|  | Same |  | 154 (35.9) |  | 40 (35.7) | 70 (36.1) | 39 (36.8) |  |
|  | Agree |  | 154 (35.9) |  | 49 (43.8) | 67 (34.5) | 34 (32.1) |  |
| I have improved my sleep habits | |  |  |  |  |  |  |  |
|  | Disagree |  | 2297 (30.1) |  | 411 (40.8) | 928 (29.7) | 857 (28.0) | 1.E-16 |
|  | Same |  | 4170 (54.7) |  | 475 (47.2) | 1797 (57.5) | 1617 (52.9) |  |
|  | Agree |  | 1159 (15.2) |  | 121 (12.0) | 401 (12.8) | 585 (19.1) |  |
| I have improved my nutrition (Dietary Habits) | |  |  |  |  |  |  |  |
|  | Disagree |  | 1780 (23.1) |  | 291 (28.6) | 751 (23.7) | 663 (21.6) | 7.E-05 |
|  | Same |  | 3914 (50.8) |  | 490 (48.1) | 1599 (50.5) | 1567 (51.0) |  |
|  | Agree |  | 2007 (26.1) |  | 238 (23.4) | 817 (25.8) | 843 (27.4) |  |
| I have gained weight | |  |  |  |  |  |  |  |
|  | Disagree |  | 2486 (32.4) |  | 308 (30.5) | 1004 (31.8) | 1017 (33.2) | 0.027 |
|  | Same |  | 2743 (35.7) |  | 331 (32.8) | 1162 (36.7) | 1078 (35.1) |  |
|  | Agree |  | 2454 (31.9) |  | 371 (36.7) | 996 (31.5) | 972 (31.7) |  |
| How concerned have you been about the novel COVID-19 pandemic in the past 7 days? | |  |  |  |  |  |  |  |
|  |  |  | 5.57 (2.95) |  | 5.61 (3.11) | 5.60 (2.99) | 5.49 (2.86) | 0.298 |
| How concerned are you about - Contracting COVID-19 | |  |  |  |  |  |  |  |
|  | Not-to-slightly concerned |  | 4162 (52.6) |  | 535 (50.7) | 1676 (51.6) | 1727 (55.1) | 0.006 |
|  | Very-to-extremely concerned |  | 3747 (47.4) |  | 520 (49.3) | 1573 (48.4) | 1410 (44.9) |  |
| How concerned are you about - Someone close to you contracting COVID-19 | |  |  |  |  |  |  |  |
|  | Not-to-slightly concerned |  | 2983 (37.7) |  | 402 (38.1) | 1233 (38.0) | 1184 (37.7) | 0.973 |
|  | Very-to-extremely concerned |  | 4926 (62.3) |  | 653 (61.9) | 2016 (62.0) | 1953 (62.3) |  |
| How concerned are you about - Getting into serious financial trouble | |  |  |  |  |  |  |  |
|  | Not-to-slightly concerned |  | 6461 (81.7) |  | 689 (65.3) | 2587 (79.6) | 2767 (88.2) | 1.E-58 |
|  | Very-to-extremely concerned |  | 1448 (18.3) |  | 366 (34.7) | 662 (20.4) | 370 (11.8) |  |
| How concerned are you about - Losing your job | |  |  |  |  |  |  |  |
|  | Not-to-slightly concerned |  | 7071 (89.4) |  | 910 (86.3) | 2879 (88.6) | 2846 (90.7) | 1.E-04 |
|  | Very-to-extremely concerned |  | 838 (10.6) |  | 145 (13.7) | 370 (11.4) | 291 ( 9.3) |  |
| How concerned are you about - That it will be a long time before your life returns to normal | |  |  |  |  |  |  |  |
|  | Not-to-slightly concerned |  | 3841 (48.6) |  | 474 (44.9) | 1581 (48.7) | 1551 (49.4) | 0.038 |
|  | Very-to-extremely concerned |  | 4068 (51.4) |  | 581 (55.1) | 1668 (51.3) | 1586 (50.6) |  |
| How concerned are you about - Not seeing friends and family | |  |  |  |  |  |  |  |
|  | Not-to-slightly concerned |  | 3673 (46.4) |  | 471 (44.6) | 1496 (46.0) | 1515 (48.3) | 0.063 |
|  | Very-to-extremely concerned |  | 4236 (53.6) |  | 584 (55.4) | 1753 (54.0) | 1622 (51.7) |  |
