# Supplementary material for: Exposure and risk factors for COVID-19 and the impact of staying home on Michigan residents
Source: PLoS One. 2021 Feb 8;16(2):e0246447. doi: 10.1371/journal.pone.0246447 (PMC7870003; doi:10.1371/journal.pone.0246447)
Supplement: S10 Table — (DOCX) [file pone.0246447.s010.docx]

| **Table S10.** Behavioral change by race | | | | | | | |
| --- | --- | --- | --- | --- | --- | --- | --- |
|  |  |  | **Overall** |  | **Race** | |  |
|  |  |  |  |  | **AA** | **EA** | **p** |
|  |  | n | 7909 |  | 220 | 7279 |  |
| I have increased moderate to strenuous exercise | |  |  |  |  |  |  |
|  | Disagree |  | 2921 (38.6) |  | 92 (44.7) | 2669 (38.3) | 0.485 |
|  | Same |  | 2907 (38.4) |  | 59 (28.6) | 2710 (38.9) |  |
|  | Agree |  | 1744 (23.0) |  | 55 (26.7) | 1596 (22.9) |  |
| I have increased my alcohol consumption | |  |  |  |  |  |  |
|  | Disagree |  | 3530 (52.7) |  | 98 (58.0) | 3241 (52.3) | 0.248 |
|  | Same |  | 1949 (29.1) |  | 40 (23.7) | 1818 (29.3) |  |
|  | Agree |  | 1219 (18.2) |  | 31 (18.3) | 1136 (18.3) |  |
| I have increased my drug use | |  |  |  |  |  |  |
|  | Disagree |  | 854 (68.3) |  | 23 (62.2) | 785 (68.8) | 0.446 |
|  | Same |  | 237 (19.0) |  | 9 (24.3) | 215 (18.8) |  |
|  | Agree |  | 159 (12.7) |  | 5 (13.5) | 141 (12.4) |  |
| I have increased my tobacco use | |  |  |  |  |  |  |
|  | Disagree |  | 121 (28.2) |  | 5 (25.0) | 109 (28.4) | 0.930 |
|  | Same |  | 154 (35.9) |  | 8 (40.0) | 135 (35.2) |  |
|  | Agree |  | 154 (35.9) |  | 7 (35.0) | 140 (36.5) |  |
| I have improved my sleep habits | |  |  |  |  |  |  |
|  | Disagree |  | 2297 (30.1) |  | 88 (40.9) | 2085 (29.7) | 0.004 |
|  | Same |  | 4170 (54.7) |  | 96 (44.7) | 3877 (55.2) |  |
|  | Agree |  | 1159 (15.2) |  | 31 (14.4) | 1060 (15.1) |  |
| I have improved my nutrition (Dietary Habits) | |  |  |  |  |  |  |
|  | Disagree |  | 1780 (23.1) |  | 58 (26.9) | 1633 (23.0) | 0.224 |
|  | Same |  | 3914 (50.8) |  | 83 (38.4) | 3651 (51.5) |  |
|  | Agree |  | 2007 (26.1) |  | 75 (34.7) | 1808 (25.5) |  |
| I have gained weight | |  |  |  |  |  |  |
|  | Disagree |  | 2486 (32.4) |  | 64 (30.2) | 2298 (32.5) | 0.379 |
|  | Same |  | 2743 (35.7) |  | 75 (35.4) | 2522 (35.7) |  |
|  | Agree |  | 2454 (31.9) |  | 73 (34.4) | 2251 (31.8) |  |
| How concerned have you been about the novel COVID-19 pandemic in the past 7 days? | |  |  |  |  |  |  |
|  |  |  | 5.57 (2.95) |  | 6.74 (3.01) | 5.53 (2.94) | 2.E-09 |
| How concerned are you about - Contracting COVID-19 | |  |  |  |  |  |  |
|  | Not-to-slightly concerned |  | 4162 (52.6) |  | 80 (36.4) | 3857 (53.0) | 2.E-06 |
|  | Very-to-extremely concerned |  | 3747 (47.4) |  | 140 (63.6) | 3422 (47.0) |  |
| How concerned are you about - Someone close to you contracting COVID-19 | |  |  |  |  |  |  |
|  | Not-to-slightly concerned |  | 2983 (37.7) |  | 62 (28.2) | 2760 (37.9) | 0.004 |
|  | Very-to-extremely concerned |  | 4926 (62.3) |  | 158 (71.8) | 4519 (62.1) |  |
| How concerned are you about - Getting into serious financial trouble | |  |  |  |  |  |  |
|  | Not-to-slightly concerned |  | 6461 (81.7) |  | 145 (65.9) | 6006 (82.5) | 9.E-10 |
|  | Very-to-extremely concerned |  | 1448 (18.3) |  | 75 (34.1) | 1273 (17.5) |  |
| How concerned are you about - Losing your job | |  |  |  |  |  |  |
|  | Not-to-slightly concerned |  | 7071 (89.4) |  | 175 (79.5) | 6544 (89.9) | 1.E-06 |
|  | Very-to-extremely concerned |  | 838 (10.6) |  | 45 (20.5) | 735 (10.1) |  |
| How concerned are you about - That it will be a long time before your life returns to normal | |  |  |  |  |  |  |
|  | Not-to-slightly concerned |  | 3841 (48.6) |  | 91 (41.4) | 3545 (48.7) | 0.032 |
|  | Very-to-extremely concerned |  | 4068 (51.4) |  | 129 (58.6) | 3734 (51.3) |  |
| How concerned are you about - Not seeing friends and family | |  |  |  |  |  |  |
|  | Not-to-slightly concerned |  | 3673 (46.4) |  | 93 (42.3) | 3384 (46.5) | 0.217 |
|  | Very-to-extremely concerned |  | 4236 (53.6) |  | 127 (57.7) | 3895 (53.5) |  |
